# Advances in Leukemia detection and classification: A Systematic review of AI and image processing techniques

**DOI:** 10.12688/f1000research.159318.2

**Published:** 2025-10-02

**Authors:** Aya Achir, Ikram Debbarh, Nadia Zoubir, Ilham Battas, Hicham Medromi, Fouad Moutaouakkil

**Affiliations:** 1 Research Foundation for Research Development and Innovation in Science and Engineering, Casablanca, Grand Casablanca, Morocco; 2 Engineering Research Laboratory (LRI), National High School of Electricity and Mechanic (ENSEM), University Hassan II Casablanca, Casablanca, Grand Casablanca, Morocco; 3 The International Academy of Scientific Francophonie (AIFS), Rabat, Morocco

**Keywords:** Leukemia Classification, Artificial Intelligence in Medical Diagnostics, Machine Learning Algorithms, Hematologic Malignancies, Deep Learning Models, Convolutional Neural Networks, Cancer Epidemiology, Diagnostic Accuracy.

## Abstract

**Background:**

Leukemia, a heterogeneous group of blood cancers, poses significant challenges to global health due to its complexity, diverse risk factors, and variable outcomes. Accurate and early diagnosis is critical but remains a significant hurdle, particularly in low-resource settings. Recent advancements in artificial intelligence (AI) and image processing offer transformative solutions to improve leukemia detection and classification, addressing limitations in traditional diagnostic methods.

**Methods:**

This study systematically reviewed over 25,000 scientific articles sourced from Scopus, employing a PRISMA-guided methodology to ensure a comprehensive and rigorous analysis. The analysis focused on the application of AI, particularly convolutional neural networks (CNNs), in diagnosing four primary leukemia types: acute lymphoblastic leukemia (ALL), acute myeloid leukemia (AML), chronic lymphocytic leukemia (CLL), and chronic myeloid leukemia (CML). It also examined global epidemiological trends, risk factors, and disparities in healthcare access.

**Results:**

Key risk factors for leukemia include genetic syndromes like Down syndrome, environmental exposures to toxins such as benzene, ionizing radiation, and viral infections. Socio-economic disparities and geographical differences significantly impact leukemia incidence and outcomes. AI-based models, especially CNNs, demonstrated enhanced accuracy, speed, and reliability in diagnosing leukemia compared to traditional methods. However, challenges such as data variability, model scalability, and unequal access to AI technologies continue to hinder widespread adoption.

**Conclusion:**

AI and image processing technologies hold immense potential to revolutionize leukemia diagnostics by enabling early detection, precise classification, and personalized treatment planning. Addressing critical challenges, including data standardization and equitable access to these technologies, will be vital for global application. This review highlights the transformative role of AI in improving leukemia outcomes and advancing precision medicine worldwide.

## 1. Introduction

Leukemia is a complex hematological malignancy defined by the abnormal proliferation of white blood cells, which disrupts the body’s blood-forming tissues and interferes with normal immune functions. This group of cancers encompasses both acute and chronic forms affecting either lymphoid or myeloid cells, resulting in various subtypes with distinct clinical features and progression rates. ^1, 2^ Key characteristics of leukemia include rapid, uncontrolled cell growth that impairs immune functions, leads to anemia, and creates other systemic impacts. Leukemia’s morphological diversity complicates diagnosis and subtype differentiation, as highlighted in recent classification studies. ^3^ This diversity in presentation underscores the need for accurate classification, which is essential for effective prognosis and treatment planning.

Due to high mortality rates and diagnostic challenges associated with leukemia, accurate classification has become increasingly critical. Advances in artificial intelligence (AI) are transforming leukemia detection and classification in medical imaging. Studies demonstrate that machine learning and deep learning models, including convolutional neural networks (CNNs), enhance diagnostic precision and enable faster subtype identification, promising substantial potential for clinical applications. ^4^^,^^5^ This study aims to address the specific challenges in leukemia classification by developing an AI-driven approach to accurately detect and classify leukemia subtypes.

This paper provides an in depth exploration of leukemia detection and classification, beginning with an analysis of global epidemiology to outline incidence, prevalence, and risk factors across regions and demographics, thereby highlighting disparities and risk factors. The second section delves into classification, focusing on primary leukemia subtypes: ALL, AML, CLL, and CML and examining morphological and genetic characteristics important for diagnosis. In the third section, we describe our methodology, which details our data collection, processing, and analytical techniques used to perform a robust bibliometric analysis. This bibliometric analysis identifies publication trends, key contributors, and influential studies, offering insights into the current landscape of leukemia research. Lastly, we present a comparison of state-of-the-art AI applications for leukemia, reviewing machine learning and deep learning techniques employed in diagnostics. Here, we discuss each approach’s limitations, emphasizing challenges related to data quality, generalizability, and integration into clinical workflows.

## 2. Global epidemiology and risk factors of Leukemia

The epidemiology of leukemia has been extensively analyzed through various studies over the years, revealing significant trends, risk factors, and geographical disparities. Bouchbika et al. conducted a study in Greater Casablanca from 2005 to 2007, using data from the Casablanca cancer registry. They found age-standardized rates (ASR) of 2.7 per 100,000 for men and 2.0 for women, with incidence peaks in children aged 59 and adults over 65. Comparatively, Morocco’s leukemia rates were lower than those in Tunisia and Algeria, and significantly lower than Western countries. Childhood leukemia (0-14 years) constituted 10.9% of all childhood cancers with an ASR of 1.4. ^6^ Expanding the analysis globally, Miranda-Filho et al. used data from 290 cancer registries in 68 countries and national estimates from 2012. Their study found the highest ASRs in Australia, New Zealand (11.3 per 100,000 in males, 7.2 in females), Northern America (10.5 in males, 7.2 in females), and Western Europe (9.6 in males, 6.0 in females). Conversely, Western Africa had the lowest rates (1.4 in males, 1.2 in females). Acute lymphoblastic leukemia was predominant in children, while chronic lymphocytic leukemia was more common in adults in European and North American countries. Key risk factors identified included genetic syndromes such as Down syndrome, environmental exposures like benzene and ionizing radiation, and viral infections. ^7^ In Arab countries, Al-Muftah and Al-Ejeh highlighted higher leukemia incidence rates among younger populations, particularly in the Arabian Gulf and Levant regions. Utilizing GLOBOCAN data from 2003 to 2016, the study noted elevated age-standardized incidence rates (ASIR) in children under 15 years compared to global averages. For adults aged 35 and older, the Levant region also showed higher rates than the global norm. These findings emphasized the need for targeted genetic epidemiology studies and improved clinical management strategies. ^8^ Further, ElBakali, Abdellatif, and Smiri examined 8,851 cancer cases in the Souss Massa region of Morocco from 2014 to 2019. Hematological cancers, including leukemia, accounted for 14% of cases, with a female incidence rate of 47.74 per 100,000 and a male incidence rate of 45.71 per 100,000. The average age of leukemia patients was 47.81 years. Their analysis underscored the importance of reinforcing prevention efforts and developing specific screening strategies for hematological cancers. ^9^ Baeker Bispo, Pinheiro, and Kobetz provided a comprehensive overview of leukemia and lymphoma in the US and globally, reporting higher incidence rates in developed regions and racial disparities in survival rates. They noted that leukemia is the 15th most common cancer worldwide, accounting for 437,033 cases and 309,006 deaths in 2018. The study highlighted genetic abnormalities, immunosuppression, ionizing radiation, carcinogenic chemicals, and oncogenic viruses as key risk factors. They emphasized the need for equitable access to diagnostic and treatment services to address these disparities. ^10^ Smith Torres-Roman et al. analyzed leukemia motality trends in children from 15 Latin American countries between 2000 and 2017. They found the highest mortality rates in Venezuela, Ecuador, Nicaragua, Mexico, and Peru, with significant upward trends in Nicaragua and Peru. Conversely, Puerto Rico saw substantial declines. The study predicted that by 2030, leukemia mortality would increase in several countries, emphasizing the need for interventions to reduce inequalities and ensure universal healthcare coverage. ^11^ Huang et al. conducted a global analysis using data from GLOBOCAN, CI5, WHO, NORDCAN, and SEER databases. They found that leukemia accounted for 2.5% of new cancer cases and 3.1% of cancer deaths, with an age-standardized incidence rate (ASIR) of 5.4 and mortality rate of 3.3 per 100,000 people. The study associated higher incidence and mortality rates with the Human Development Index, GDP per capita, and lifestyle factors such as smoking and obesity. They recommended lifestyle modifications and further research to understand these trends. ^12^ Finally, Zhang et al. examined the global burden of hematologic malignancies from 1990 to 2019 using data from the Global Burden of Disease (GBD) study. They found a declining trend in leukemia incidence (ASIR of 8.22 per 100,000) and mortality (ASDR of 4.26 per 100,000), with significant decreases in age-standardized rates.

However, regions like Central Europe, Western Europe, and East Asia experienced increases. The study highlighted the influence of socio-economic factors, occupational exposures, and high BMI on leukemia burden, emphasizing the need for targeted prevention strategies and improved healthcare access. ^13^ these studies collectively highlight the global variations in leukemia incidence and mortality, influenced by genetic, environmental, and socio-economic factors. While some regions have seen declining trends, others continue to face significant challenges, underscoring the need for comprehensive prevention and management strategies tailored to specific regional needs.

## 3. Classification of Leukemia

Historically, leukemias were classified using the French–American–British (FAB) system based on morphology (e.g. AML M0–M7 and ALL L1–L3). However, this purely morphological classification is now obsolete and provides limited prognostic or therapeutic guidance. ^3^ The current standard is to use the **WHO 5th Edition (2022)** and the **International Consensus Classification (ICC, 2022),** which integrate morphology with immunophenotypic and genetic features. These modern frameworks emphasize clinically actionable biomarkers and prognostic factors, ^14^ leading to an evidence-based, globally applicable classification of leukemias and related neoplasms. In the WHO-HAEM5 and ICC systems, hematologic malignancies are organized by **cell lineage** (myeloid vs lymphoid) and by the nature of the disease (acute vs chronic). The term *“leukemia”* is not a single category but rather spans multiple distinct entities across these classifications. Broadly, the major categories include ^3^:

• **Myeloid Neoplasms:** This group encompasses acute myeloid leukemias (AML) as well as chronic myeloproliferative neoplasms. For example, **Chronic Myeloid Leukemia (CML)** is classified as a myeloproliferative neoplasm (MPN) characterized by the BCR-ABL1 fusion gene. The MPN category also includes disorders such as Polycythemia Vera, Essential Thrombocythemia, Primary Myelofibrosis, and less common entities like Chronic Neutrophilic Leukemia (CNL) and chronic eosinophilic leukemia. ^3^^,^^15^ There are also overlap syndromes (myelodysplastic/myeloproliferative neoplasms like **Chronic Myelomonocytic Leukemia, CMML**) and neoplasms defined by specific mutations (e.g. myeloid/lymphoid neoplasms with eosinophilia and tyrosine kinase gene fusions). AML itself is no longer defined solely by morphology but by distinct subtypes with recurrent genetic abnormalities or clinical contexts (such as *AML with t(8;21)*, *AML with biallelic CEBPA mutations*, or therapy-related AML), replacing the old FAB M0–M7 nomenclature. ^3^
• **Lymphoid Neoplasms:** This includes **precursor lymphoid neoplasms** (acute lymphoblastic leukemias/lymphomas) and **mature (post-germinal center) lymphoid neoplasms. Acute Lymphoblastic Leukemia (ALL)** is classified as either B-lymphoblastic or T-lymphoblastic leukemia/lymphoma, with subgroups defined by specific cytogenetic or molecular alterations rather than FAB L1–L3 morphology. ^16^

In contrast, Chronic Lymphocytic Leukemia typically denoted as CLL/SLL (for CLL/Small Lymphocytic Lymphoma) is a mature B-cell neoplasm. WHO-HAEM5 places CLL/SLL in the family of small B-cell lymphoid proliferations alongside its precursor, Monoclonal B-cell Lymphocytosis (MBL), and notably eliminates B-cell Prolymphocytic Leukemia (B-PLL) as an independent entity. ^16^ (Cases formerly called “B-PLL” are now understood to be heterogeneous; depending on their features, they are reclassified as a leukemic variant of mantle cell lymphoma, as CLL in prolymphocytic phase, or as other specific entities. ^16^)

**Figure 1.**
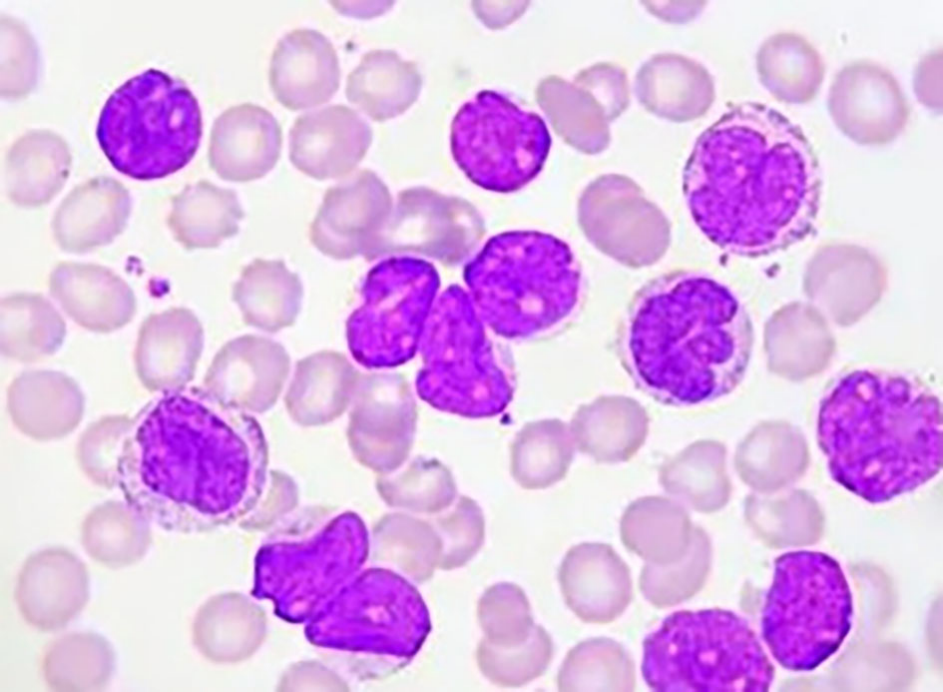
Microscopic image of Acute Lymphoblastic Leukemia.

**Figure 2.**
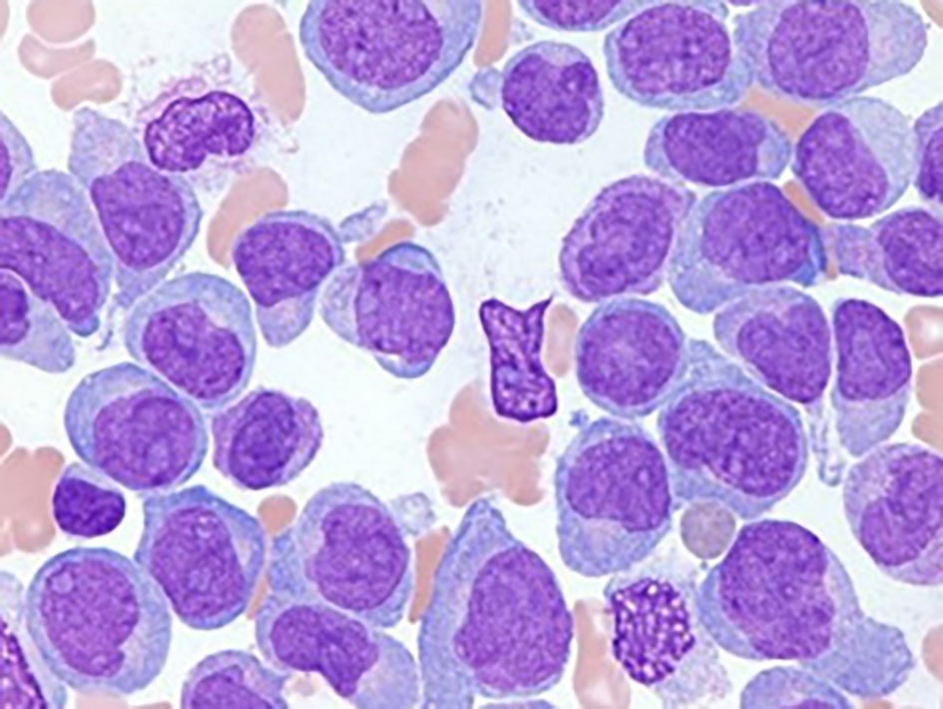
Microscopic image of Acute Myeloid Leukemia.

**Figure 3.**
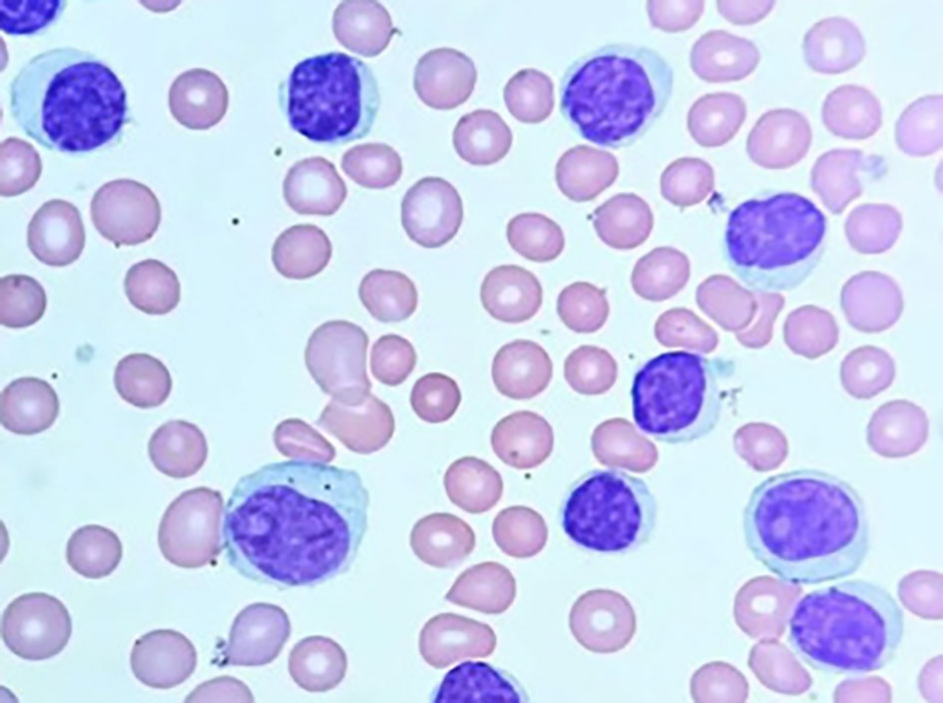
Microscopic image of Chronic Lymphocytic Leukemia.

**Figure 4.**
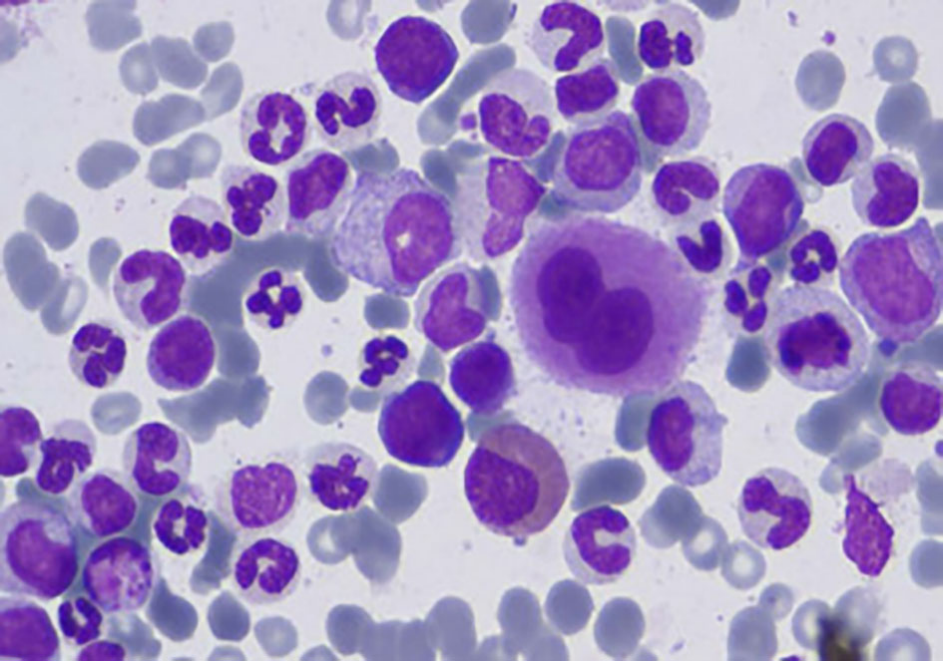
Microscopic image of Chronic Myeloid Leukemia.

Other mature B-cell neoplasms that often present with leukemic involvement include mantle cell lymphoma (which can have a leukemic phase), Hairy Cell Leukemia, splenic marginal zone lymphoma, and the recently defined splenic B-cell leukemia/lymphoma with prominent nucleoli (encompassing what was known as HCL-variant and some cases of former B-PLL). ^16^ Similarly, there are chronic T- or NK-cell leukemias such as T-Prolymphocytic Leukemia (T-PLL), T large granular lymphocytic leukemia (T-LGLL), NK-LGLL, Adult T-cell leukemia/lymphoma (ATLL), Sézary syndrome, and Aggressive NK-cell leukemia, all of which are defined as distinct entities in the modern classification. ^16^ These diseases, while bearing unique names, can present with peripheral blood involvement (a “leukemic” phase), and thus fall under the broad umbrella of leukemic lymphoid neoplasms in practice. Figure 1 shows Acute Lymphoblastic Leukemia (ALL) cells, Figure 2 depicts Acute Myeloid Leukemia (AML) cells, Figure 3 illustrates Chronic Lymphocytic Leukemia (CLL) cells, and Figure 4 presents Chronic Myeloid Leukemia (CML) cells, each highlighting their distinct morphological features.

Table 1 presents an overview of the major leukemia subtypes within the 2022 WHO and ICC classification frameworks, highlighting how acute and chronic leukemias are distributed among the myeloid and lymphoid categories of disease. This updated classification approach ensures that each “leukemia” is identified in a precise context (defined by lineage, cell maturity, and genetic features) rather than being grouped by morphology alone.

**Table 1.** WHO5 and ICC (2022) classification of major leukemias and related entities.

Entity	WHO5/ICC c category	Key defining features	Notes
Acute Lymphoblastic Leukemia (ALL)	Precursor lymphoid neoplasm	B-ALL or T-ALL; subclassified by genetic lesions (e.g., BCR::ABL1, ETV6::RUNX1, KMT2A); FAB L3 now classified as Burkitt lymphoma/leukemia	Risk and therapy guided by genetics (e.g., TKIs for Ph+ ALL)
Acute Myeloid Leukemia (AML)	Myeloid neoplasm	Defined by recurrent genetic abnormalities (e.g., RUNX1::RUNX1T1, CBFB::MYH11, PML::RARA, NPM1, CEBPA, TP53); blast % criteria adjusted by WHO5/ICC	Genetic subtype supersedes morphology; informs therapy (e.g., ATRA/arsenic for APL)
Chronic Lymphocytic Leukemia (CLL/SLL)	Mature B-cell neoplasm	Clonal small B-cells (CD5+, CD23+, dim CD20/Ig); ≥5×10 ^9^/L in blood = CLL; <5×10 ^9^/L + nodal disease = SLL	No morphologic subtypes; prognosis guided by IGHV & TP53 status; other leukemic B/T neoplasms include mantle cell, HCL, SMZL, T-PLL, T-/NK-LGLL
Chronic Myeloid Leukemia (CML)	Myeloproliferative neoplasm (MPN)	Defined by BCR::ABL1 fusion; phases: chronic, accelerated, blast	Prototype of targeted therapy (TKIs); differentials include CNL, CMML, atypical CML, eosinophilia with TK fusions

As an example of how modern classification has evolved, consider the case of Burkitt leukemia. The FAB scheme designated Burkitt-type acute lymphoblastic leukemia as “L3 ALL.” In current WHO/ICC classifications, this is no longer viewed as a subtype of ALL it is recognized as **Burkitt Lymphoma/Leukemia**, a mature B-cell neoplasm with a characteristic MYC translocation. ^17^ In other words, cases that would previously be called L3 ALL are now classified and treated as **Burkitt lymphoma**, reflecting their mature B-cell origin. More generally, both WHO-5 and ICC classify **B-ALL** and **T-ALL** into multiple subgroups defined by cytogenetics (for example, **B-ALL with t(9;22)(q34;q11)/BCR-ABL1**, **B-ALL with KMT2A rearrangement**, **T-ALL with TLX3 rearrangement**, etc.), ^16^ and they align on many criteria for diagnosis and risk stratification in acute leukemias. For instance, the essential immunophenotypic markers for diagnosing CLL/SLL (CD19, CD20, CD5, CD23, etc.) are the same in both WHO and ICC, and both systems recommend genomic analyses such as IGHV mutation status and TP53 deletion/mutation status for prognostication in CLL. ^18^ This convergence underscores that the two classification frameworks are largely consistent in defining entities, even if some nomenclature differs.

In summary, the classification of leukemia in the 5th WHO edition and ICC is a modern, integrated scheme that supersedes the older FAB morphology-based taxonomy. ^3^ By incorporating immunophenotypic and molecular criteria, the WHO/ICC classifications better reflect the biology of the disease and have greater relevance to patient management. ^14^ This means that the term “leukemia” is applied with specificity – distinguishing, for example, a **chronic myeloid leukemia (an MPN with a particular tyrosine kinase fusion)** from a **B-lymphoblastic leukemia (an acute lymphoid neoplasm defined by lineage and genotype)** or from **chronic lymphocytic leukemia (a mature B-cell lymphoma/leukemia)**. Such precision in classification enhances prognostication and guides therapy, ensuring that each patient’s leukemia is categorized in a way that informs optimal care. ^14^

In conclusion, beyond molecular refinements in classification, emerging computational methods such as microscopic image-based analysis are proving effective in distinguishing leukemic subtypes. ^19^ These advances provide a natural bridge to the next section, which explores the broader role of artificial intelligence in medical diagnostics.

## 4. Overview of AI in medical diagnostics

world of medical diagnostics, offering a powerful set of tools to analyze and interpret vast amounts of medical data. ^18^ Subfields of AI, like machine learning (ML) and deep learning (DL), are particularly adept at finding patterns and making predictions based on these large datasets. In healthcare, this translates to several key benefits: improved diagnostic accuracy, better prediction of patient outcomes, and the potential for personalized treatment plans. Machine learning algorithms, such as support vector machines (SVMs) and k-nearest neighbors (KNNs), have proven successful in diagnosing diseases. These algorithms can analyze medical images and patient data to identify patterns indicative of specific conditions. ^20^ Deep learning, particularly convolutional neural networks (CNNs), has become a game-changer in medical imaging. CNNs are exceptionally skilled at detecting and classifying various diseases, including cancers and cardiovascular conditions. ^21, 22^ For example, CNNs have achieved high accuracy in detecting diabetic retinopathy, show- casing their potential to revolutionize ophthalmology. ^23^ Additionally, AI-powered systems are being integrated into radiology workflows, acting as assistants to radiologists. These systems can identify abnormalities in X-rays and MRI scans, leading to fewer diagnostic errors and improved efficiency. These advancements highlight the immense potential of AI to transform healthcare delivery and ultimately improve patient care.

## 5. Methodology

In this study, we employed a comprehensive bibliometric analysis approach to explore various aspects of leukemia, leveraging data from Scopus. Our research spanned multiple queries tailored to specific sections of the study, including risk factors, classification, and the role of artificial intelligence in leukemia diagnosis and treatment. Initially, we conducted broad searches using relevant keywords in article titles, abstracts, and keywords, resulting in a cumulative total of over 25000 articles across different sections. For the section on risk factors, we identified and screened 12,218 articles, focusing on publications from 2019 to 2024. We further refined this selection based on relevance, leading to a final set of 2116 articles. The classification section included data from 12,514 articles, filtered similarly to ensure the inclusion of 2189 pertinent studies.

In our analysis of AI’s role in leukemia, we extracted data from 368 articles, highlighting key terms such as “artificial intelligence,” “machine learning,” and “deep learning.” Articles were included based on their focus on the application of AI in medical diagnostics and treatment.

We meticulously recorded detailed bibliographic information for each selected article, including authors, titles, journal names, publication dates, and citation counts. To visualize and analyze citation networks, we utilized tools like VOSviewer, which enabled us to identify central themes and emerging trends within the field. The study’s procedural flow and inclusion criteria are detailed in the PRISMA diagram, illustrating the rigorous selection and screening process undertaken to ensure a comprehensive review of the literature.

**Figure 5.**
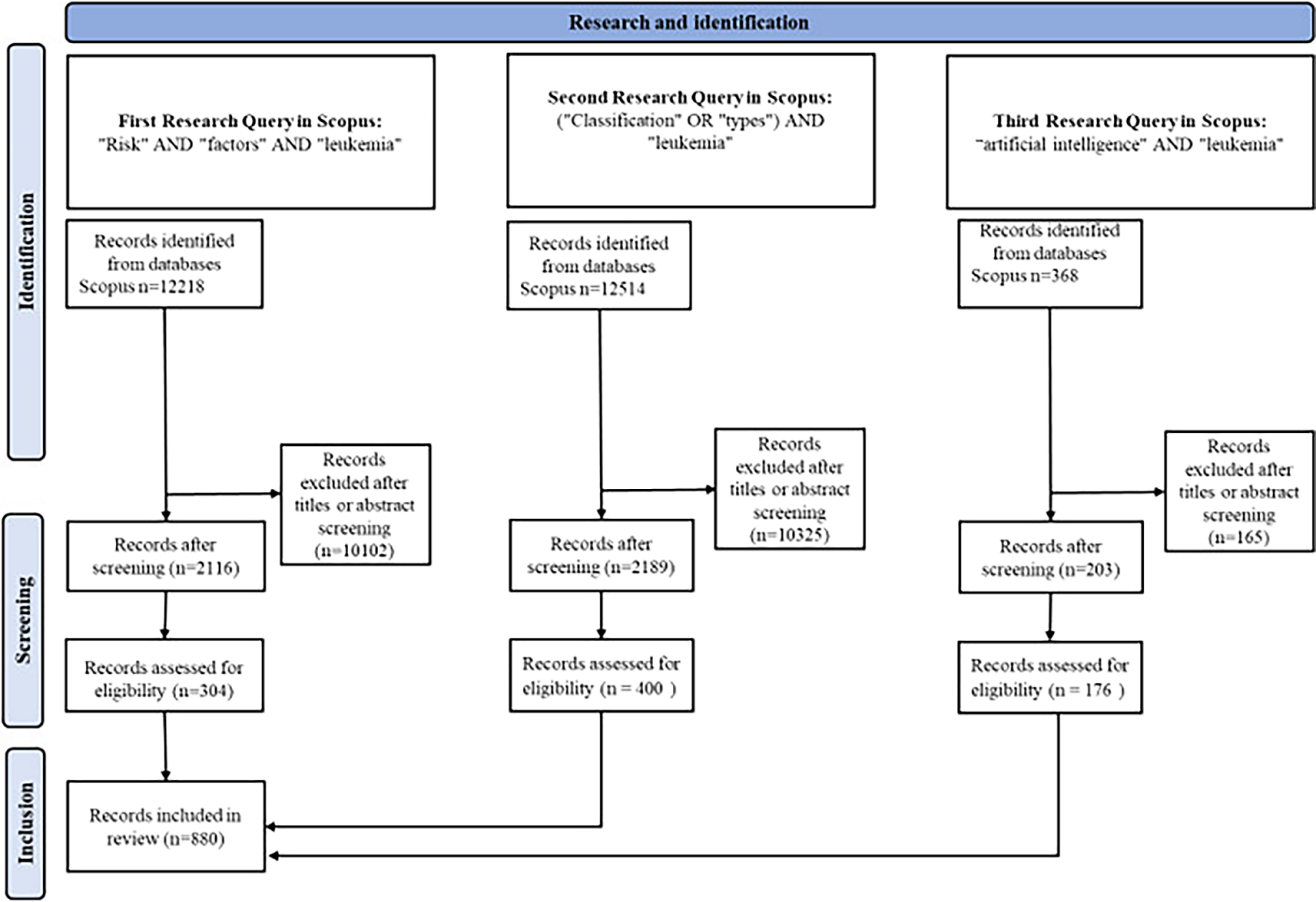
PRISMA research methodology.

Figure 5 illustrates the PRISMA research methodology used in this study.

### 5.1 Publications trend

**Figure 6.**
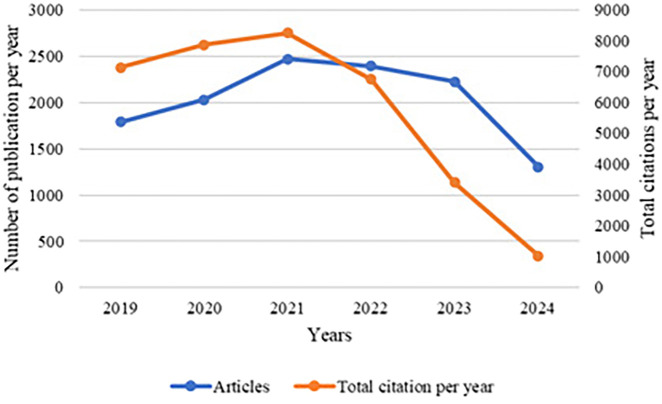
Publications trend.

Figure 6 depicts the publication trend over the years 2019 to 2024, showing the number of articles published per year and the corresponding total citations.

The graph presents the trend of publications and total citations per year related to leukemia research from 2019 to 2024. The blue line indicates the number of articles published each year, while the orange line represents the total number of citations these articles received per year.

The data reveal an initial increase in both the number of publications and citations, peaking in 2021. This suggests a heightened interest and active engagement in leukemia research during this period. The rise in publications could be attributed to advancements in research methodologies, increased funding, or a growing awareness of leukemia-related issues.

The decrease in citations from 2022 can be attributed to the time lag that often occurs in academic publishing, where newly published articles take time to be discovered, read, and subsequently cited by other researchers. This lag is a common phenomenon, as it typically takes a few years for new research to become well-known and integrated into the broader academic discourse.

### 5.2 Risk factors

**Figure 7.**
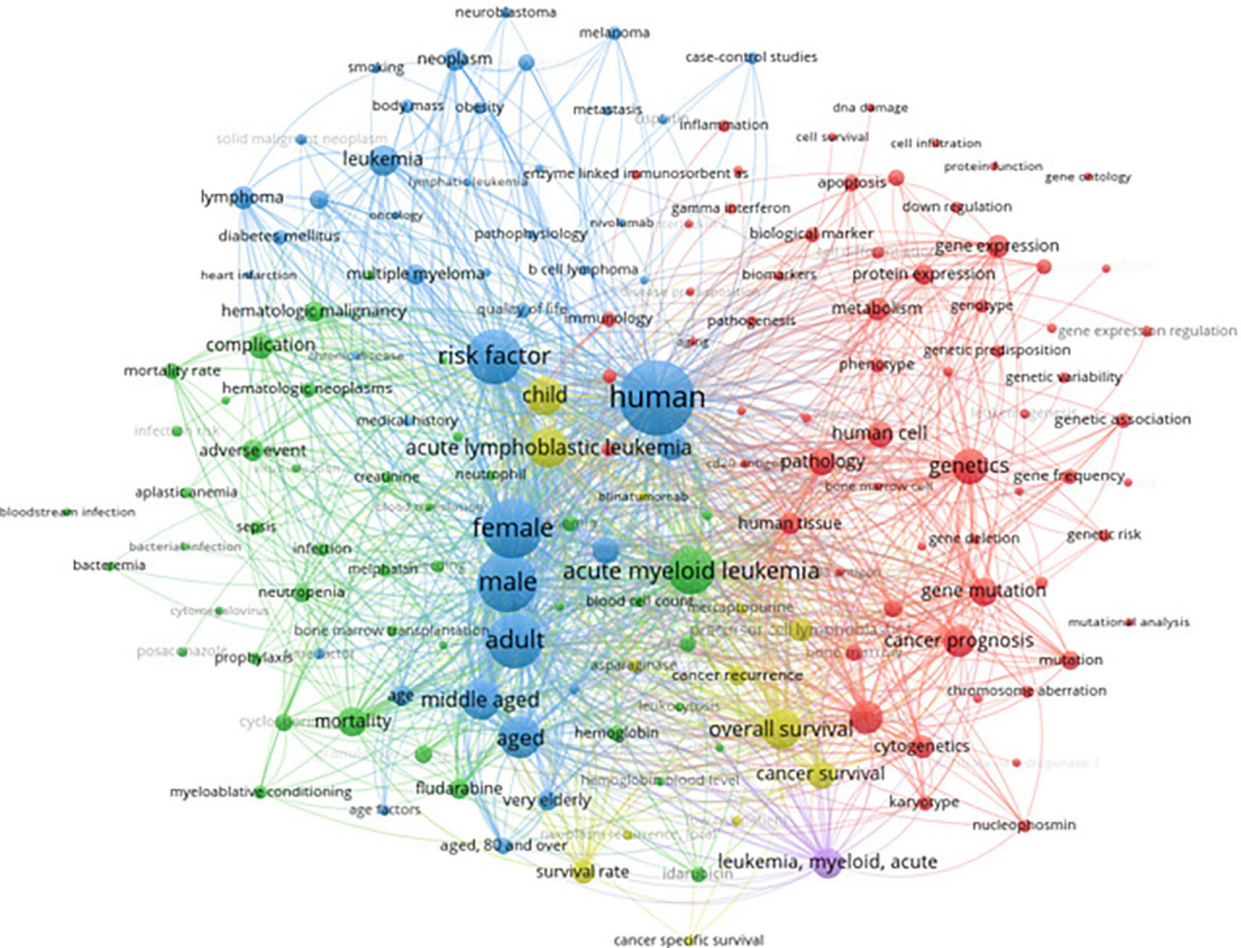
Keyword co-occurrence with risk factors.

The bibliometric analysis of leukemia risk factors ( Figure 7) revealed that recent research frequently highlights genetic predispositions, environmental exposures, and demographic influences as key themes. Notably, commonly co-occurring terms included “genetics,” “risk factor,” and specific leukemia types (e.g., acute myeloid leukemia), underscoring a research focus on how inherited mutations, toxic exposures (such as benzene or ionizing radiation), and patient characteristics (age, sex, etc.) contribute to leukemia. Indeed, the literature widely recognizes that factors like Down syndrome (a genetic syndrome), exposure to benzene or radiation, and certain viral infections can increase leukemia risk. Socio-economic and geographic disparities are also noted as influencing leukemia incidence and outcomes. This emphasis in research on etiologic risk factors provides a broad context for understanding leukemia development.

In practice, the NCCN risk stratification criteria for leukemias focus more on disease-specific and patient-specific prognostic factors than on environmental exposures. For acute leukemias (AML and ALL), the NCCN guidelines primarily stratify risk by cytogenetic and molecular abnormalities and by patient age at diagnosis. For example, in AML, NCCN (similarly to ELN) classifies patients into favorable, intermediate, or poor/adverse risk groups based on genetic features. Favorable-risk AML includes acute promyelocytic leukemia [t(15;17), PML-RARA], core-binding factor leukemias [t(8;21), inv(16)] without KIT mutations, isolated NPM1 mutations (without FLT3-ITD), and biallelic CEBPA mutations. These genetic risk categories correlate with prognosis and guide therapy: patients with favorable-risk AML have significantly better outcomes than those with adverse cytogenetics, and accordingly, NCCN recommends standard induction chemotherapy and high-dose cytarabine consolidation for favorable-risk AML, whereas intermediate- or poor-risk patients are considered for allogeneic stem cell transplant in first remission if possible. ^24^ In ALL, patient age is a critical stratifier, NCCN guidelines explicitly note that ALL treatment should be “driven in large part by patient age”. Adolescents and young adults often fare better with intensive pediatric-inspired regimens, whereas older adults (>40 or especially >60 years) may not tolerate such therapy and often have higher-risk disease features. ALL risk stratification in practice also incorporates presenting leukocyte count and cytogenetics. The NCCN guidelines call for initial stratification of ALL by the presence of the Philadelphia chromosome (BCR-ABL1); Ph-positive ALL, more common in adults, is associated with poorer prognosis and is treated with tyrosine kinase inhibitors plus chemotherapy. ^25^ Other high-risk genetic features (e.g. KMT2A/ *MLL* rearrangements, extreme hyperleukocytosis, or hypodiploidy in tumor cells) are recognized in guidelines as indications for more aggressive therapy, such as consideration of transplant in first remission. It is important to note that factors like environmental exposures or socioeconomic status, while important epidemiologically, do not appear in NCCN risk schemas for prognosis they are acknowledged as causes of leukemia but are not used to stratify patients’ risk once disease is present. For the chronic leukemias (CLL and CML), the alignment between research themes and guidelines is evident primarily in the realm of genetic prognostic markers. In CLL, our analysis highlighted the importance of genetic factors, and NCCN guidelines likewise incorporate molecular features for risk stratification and treatment selection. Specifically, the presence of a TP53 mutation or deletion 17p in CLL identifies a high-risk subset that responds poorly to standard chemoimmunotherapy. ^26^ NCCN recommendations now prioritize targeted agents (such as BTK inhibitors or BCL2 inhibitors) for these high-risk patients, ^26^ and advise against chemoimmunotherapy in cases with del(17p)/TP53 abnormalities. Even in general, the guidelines state that frontline therapy should be chosen after assessing the patient’s IGHV mutation status, del(17p)/TP53 status, age, and comorbidities. ^27^ This mirrors the literature’s focus on CLL’s biological heterogeneity: for instance, unmutated IGHV and 17p deletion are well-known adverse factors and are frequent topics in CLL research and clinical decision-making. CML differs in that its primary driver (BCR-ABL1 fusion) is present in essentially all cases, so research on “risk factors” for CML development is limited (apart from noting radiation exposure as a historical risk). Instead, both research and guidelines concentrate on prognostic scoring and response monitoring. NCCN guidelines for CML advise calculating a risk score at diagnosis such as the Sokal, Hasford (Euro), or EUTOS score – which uses factors like patient age, spleen size, and baseline blood counts (blasts, platelets, etc.) to categorize CML patients into low, intermediate, or high risk groups. ^26^ While these traditional risk scores prognosticate disease progression and help in treatment planning (for example, higher-risk CML patients might be considered for earlier allogeneic transplant if not responding optimally), it’s notable that environmental and demographic factors (e.g. prior exposure or race) do not factor into these scoring systems. In summary, the research trends identified genetics, environment, and demographics are partially reflected in clinical guidelines: genetic factors and age play a central role in risk stratification for all leukemia types in NCCN criteria (AML, ALL, CLL, CML), whereas environmental exposures and socioeconomic factors, heavily discussed in research, remain more pertinent to prevention and epidemiology than to patient risk classification in current guidelines.

Figure 7 presents a keyword co-occurrence network highlighting the relationships between terms associated with leukemia and its risk factors.

### 5.3 Types of Leukemia

The classification of leukemia was further explored through a comprehensive keyword analysis using data extracted from Scopus. This analysis encompassed 12,514 articles, focusing on keywords with more than 160 occurrences. The visualization provided a detailed mapping of the terminologies and concepts frequently associated with various types of leukemia. Prominent nodes in the network include terms like “acute myeloid leukemia,” “genetics,” “acute lymphoblastic leukemia,” and “treatment response,” among others. The graph illustrates the relationships and common themes in the literature, highlighting the intricate connections between different leukemia types, their genetic markers, treatment modalities, and related biological processes. Our findings mirror NCCN guidelines, which treat AML, ALL, CLL, and CML as distinct diseases defined by genetics and therapy response. For AML, subtypes like PML-RARA or NPM1 mutations drive targeted treatment. ^24^ In ALL, cytogenetics such as Ph-positive ALL *(*BCR-ABL1 *)* determine the need for TKIs. ^27^ CLL is stratified by molecular risks (e.g., IGHV, TP53) ^27^ while CML is classified by disease phase and BCR-ABL1 monitoring. ^28^ Across all leukemias, response metrics (CR, MRD, molecular milestones) are central in both research and NCCN practice, ensuring classification is clinically actionable.

**Figure 8.**
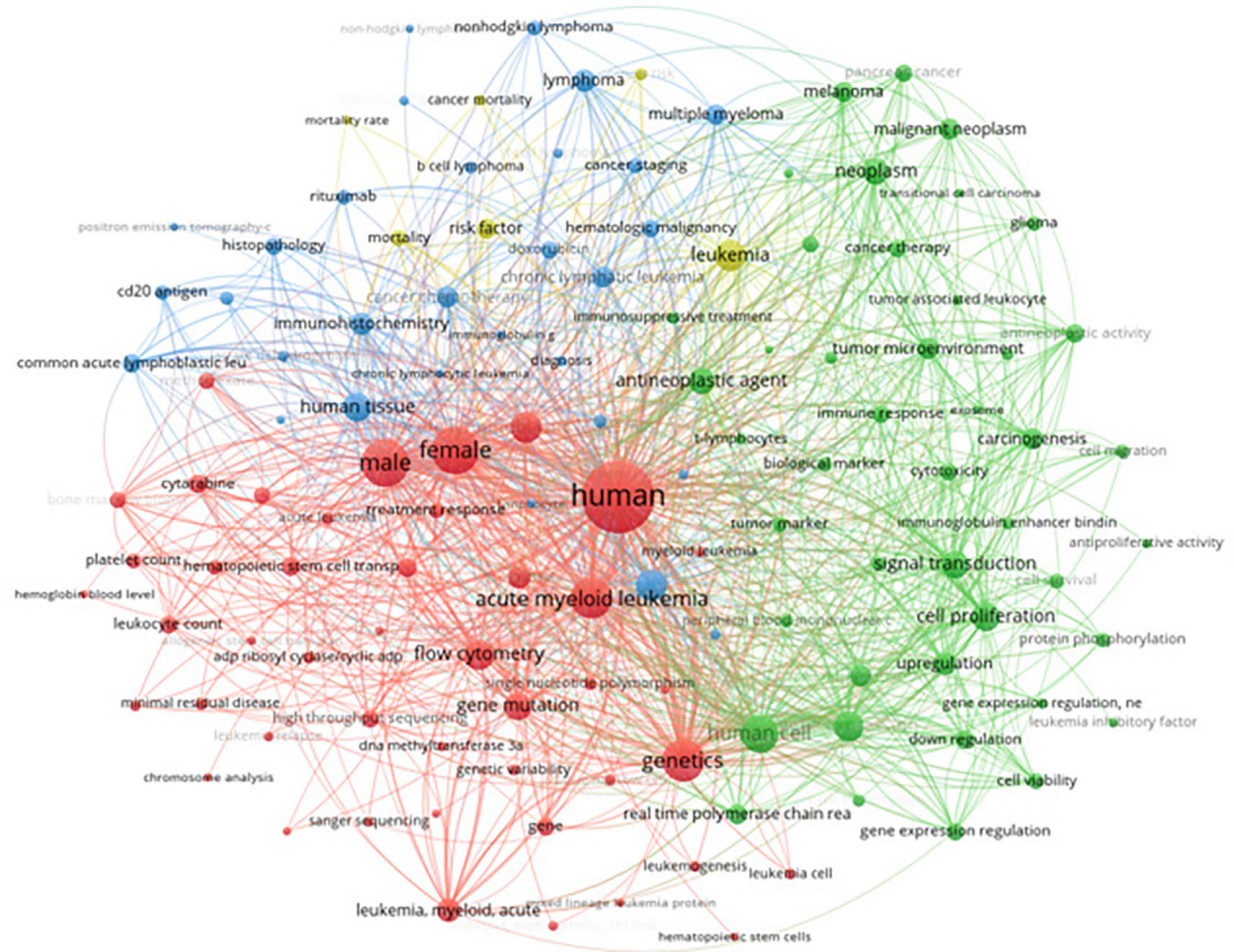
Keyword co-occurrence with classification of leukemia.

Figure 8 visualizes the keyword co-occurrence network, highlighting key terms and their relationships in the classification of leukemia.

### 5.4 The integration of AI in Leukemia research

The integration of artificial intelligence (AI) in leukemia research has been gaining significant traction, as evidenced by a comprehensive keyword analysis conducted on 368 articles from Scopus. This analysis focused on keywords with more than 20 occurrences, highlighting the prevalent themes and emerging trends within this interdisciplinary field. Key terms such as “artificial intelligence,” “machine learning,” “deep learning,” and specific medical terms like “chronic myeloid leukemia” and “diagnosis” emerged as central nodes in the network graph. The visualization showcases the increasing application of AI techniques in the medical domain, particularly in enhancing diagnostic accuracy, predicting patient outcomes, and enabling personalized treatment approaches. The centrality of AI-related keywords underscores a growing emphasis on advanced computational methods in contemporary leukemia studies.

**Figure 9.**
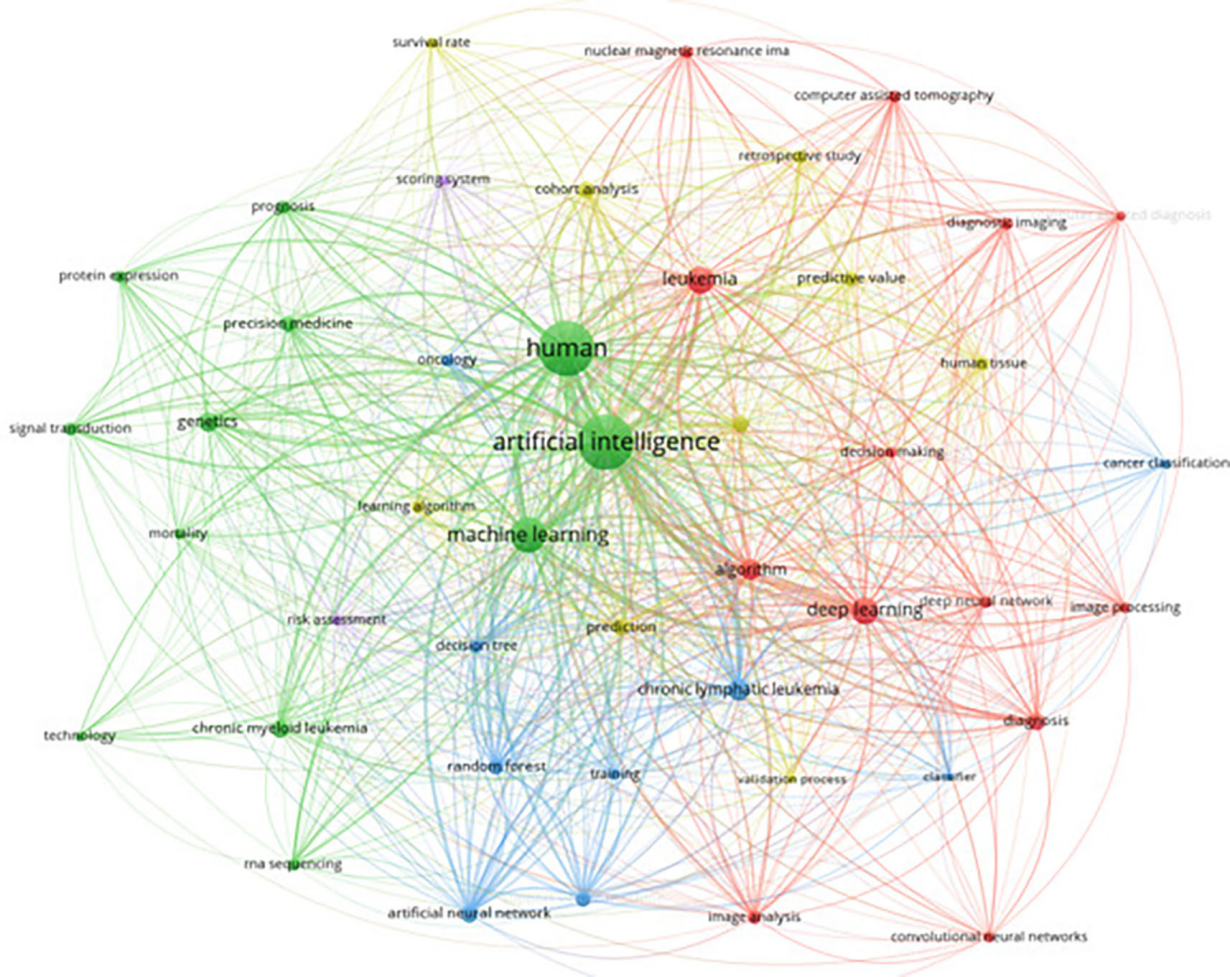
Keyword co-occurence with AI.

Figure 9 illustrates the keyword co-occurrence network focusing on AI in leukemia research.

### 5.5 Countries

Beyond keyword analysis, we investigated collaboration patterns among countries to gain a broader perspective on the global landscape of leukemia research. VOS viewer allows us to analyze” citations” as the unit of analysis, with” countries” as the collaborating entities. This analysis focused on prominent collaborations, excluding documents with a vast number of co-authoring countries. The resulting map depicts the citation impact and collaborative networks of various countries from 2016 to 2022. The visualization reveals the United States, India, and the United Kingdom as central hubs, signifying their leadership and significant contributions to leukemia research. India, in particular, displays a notable network of connections with countries like Pakistan, South Korea, and Saudi Arabia. This highlights India’s active role in fostering collaborative re- search endeavors. The color gradient, transitioning from blue to yellow, represents the publication timeline. This trend visually depicts the growing intensity of international collaborations and citations in leukemia research over the past few years. This underscores the increasingly global nature of the field and emphasizes the critical role of multinational cooperation in driving advancements in leukemia research.

**Figure 10.**
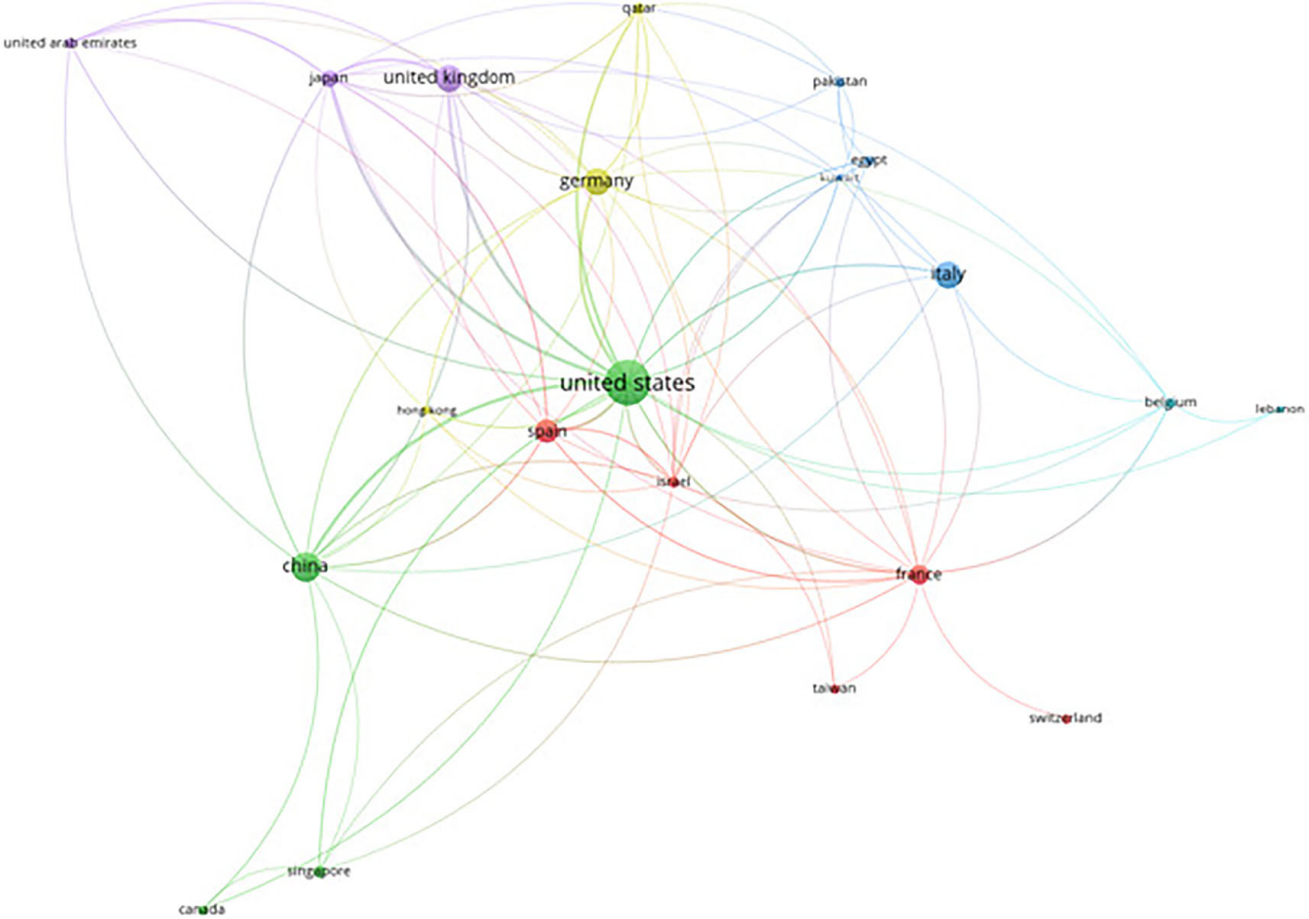
Citation analysis AI in leukemia based on countries.

Figure 10 presents a citation analysis of AI in leukemia research based on countries, highlighting the collaborative networks and the leading contributions from nations.

The bibliometric analysis revealed a clear trend: artificial intelligence (AI) is rapidly transforming leukemia research. AI offers significant advantages, including improved diagnostic accuracy, personalized treatment plans, and enhanced prediction capabilities.

These powerful techniques empower researchers and processing algorithms being applied. This in-depth examination aims to provide a clearer picture of the current landscape and identify key areas where innovation and improvement can revolutionize leukemia diagnostics and treatment.

## 6. Advancements in Leukemia detection using AI and image processing techniques

In the literature, one promising approach for leukemia detection relies on microscopic image processing through a multi-step pipeline to analyze blood smear images. The process begins with image acquisition, where high-quality blood smear images are obtained. These images then undergo preprocessing, including resizing, RGB conversion, contrast adjustment, and noise reduction, ensuring the data is optimized for further analysis. In the segmentation stage, individual cells, particularly white blood cells, are isolated from the background and neighboring cells to focus on relevant regions. The next step is feature extraction, where important characteristics like shape, size, color, texture, and morphology are identified from the segmented cells. These features are then analyzed in the leukemia detection phase, where machine learning or deep learning models process the data to identify potential abnormalities. Finally, in the classification stage, each cell is categorized as either normal or cancerous, supporting the diagnosis and treatment planning for leukemia. ^29, 30^ This structured workflow enhances accuracy by refining the data at each step, ensuring reliable detection and classification.

**Figure 11.**
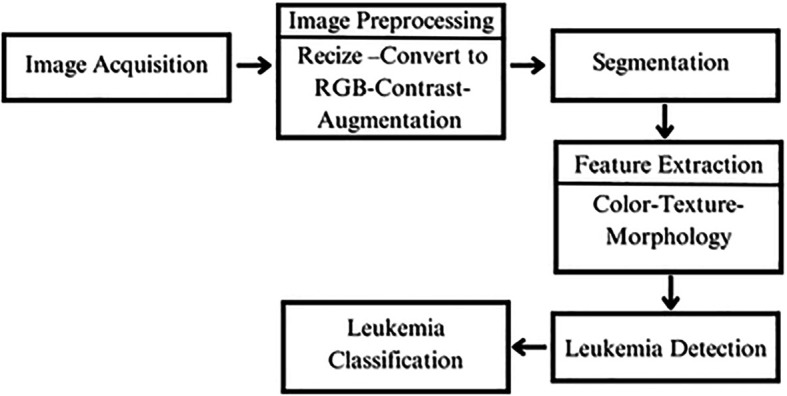
Leukemia detection workflow.

The Figure 11 illustrates the process as described in the literature.

Building on this workflow, the literature offers various innovative methodologies that further enhance the accuracy and efficiency of leukemia detection through advanced computational techniques. Recent studies demonstrate the potential of integrating machine learning, deep learning, and feature extraction methods to improve diagnostic outcomes.

Warnat-Herresthal et al. (2020) conducted a comprehensive study using 12,029 samples from 105 different studies to develop robust and scalable classifiers for Acute Myeloid Leukemia (AML). Their methodology involved rigorous preprocessing steps, including RMA normalization for microarray data and DESeq2 normalization for RNA-seq data, followed by trimming to common genes. The study employed L1-regularized logistic regression (lasso) and compared its performance with other machine learning methods such as k-nearest neighbors, linear SVM, linear discriminant analysis, random forests, and deep neural networks. The classifiers demonstrated high accuracy, sensitivity, and specificity, and were evaluated through extensive cross-study and cross-platform analyses, ensuring their robustness and generalizability. This work underscores the potential of machine learning combined with transcriptomics to enhance AML diagnostics, particularly in settings where traditional hematological expertise is limited. Despite the promising results, the study acknowledged some limitations. Performance variability due to cross-study and cross-platform differences was noted, highlighting the need for large, diverse training datasets to improve the model’s generalizability. Furthermore, the study identified the high reliance on the quality and diversity of the dataset as a potential weakness. Future directions for this research include integrating transcriptomic-based machine learning into clinical workflows for AML diagnostics, which could offer a scalable and cost-effective alternative to traditional diagnostic methods. The authors propose further refining these models and extending their application to other hematologic and non-hematologic diseases, potentially revolutionizing medical diagnostics. Additionally, future work aims to include prospective studies to assess the diagnostic utility of these models in real-world clinical settings, addressing cross-study and cross-platform variations, and exploring simple data transformations to enhance cross-platform generalizability. ^31^

Loey et al. (2020) proposed two automated classification models utilizing transfer learning with the pre-trained deep convolutional neural network, AlexNet, to classify leukemia in blood microscopic images. The first model involves pre-processing the images, extracting features using a pre-trained AlexNet, and classifying these features using various classifiers such as SVM, linear discriminants (LD), decision trees (DT), and k-nearest neighbors (K-NN). The second model fine-tunes AlexNet for both feature extraction and classification. Experiments on a dataset of 2820 images show that the second model achieves a perfect classification accuracy of 100%, outperforming the first model. The study emphasizes the advantages of using transfer learning to overcome the challenges of designing and training deep neural networks from scratch. The pre-processing step includes converting images to RGB, resizing them to 227×227 pixels, and applying data augmentation techniques like translation, reflection, and rotation. For feature extraction, AlexNet’s architecture, which includes five convolutional layers, three fully connected layers, and max-pooling layers, is utilized. The classifiers tested in the first model include decision trees with a max-split of 20, linear discriminants, SVM with various kernel functions (linear, Gaussian, cubic), and K-NN with Euclidean distance. Performance metrics such as precision, recall, accuracy, and specificity were evaluated, with the second model demonstrating higher accuracy and robustness. Despite the promising results, the study acknowledged some limitations and future directions. Potential overfitting remains a concern despite the use of dropout and normalization techniques. Additionally, the models’ dependence on high-quality, well-annotated datasets for training poses challenges for broader applicability. Future research could focus on extending these models to differentiate between various types of leukemia, rather than just identifying the presence of leukemia. Furthermore, the use of larger and more diverse datasets could help validate the models’ robustness and accuracy. The authors propose integrating these automated systems into clinical workflows to aid in the early and accurate diagnosis of leukemia, ultimately improving patient outcomes through timely and appropriate treatment. ^32^

Baig et al. (2022) developed a comprehensive approach using deep learning-based convolutional neural networks (CNN) to detect acute lymphoblastic leukemia (ALL), acute myeloid leukemia (AML), and multiple myeloma (MM) from microscopic blood smear images. The study “Detecting Malignant Leukemia Cells Using Microscopic Blood Smear Images: A Deep Learning Approach” by Raheel Baig et al. investigates an advanced automated system for detecting various types of malignant leukemia cells using deep learning techniques. The proposed methodology integrates pre-processing, feature extraction, and classification to identify Acute Lymphoblastic Leukemia (ALL), Acute Myeloid Leukemia (AML), and Multiple Myeloma (MM) from blood smear images. The dataset, comprising 4150 images, is pre-processed to enhance image quality, including background elimination, noise reduction, and contrast enhancement using adaptive histogram equalization. Two Convolutional Neural Networks (CNN-1 and CNN-2) with 19 and 15 layers, respectively, are employed for feature extraction. The extracted features are then fused using Canonical Correlation Analysis (CCA) to improve the discriminative power of the features. Finally, the fused features are classified using various machine learning classifiers, including Bagging Ensemble, LPBoost, Total Boost, RUSBoost, Fine KNN, and SVM, with the Bagging Ensemble achieving the highest accuracy of 97.04%.The methodology involves multiple steps, starting with pre-processing, where RGB images are converted to grayscale, enhanced using intensity adjustment, and noise removed through area and closing operations. Feature extraction is performed using CNN models trained on pre-processed images. CNN-1 and CNN-2, containing convolutional, batch normalization, ReLU, max-pooling, fully connected, SoftMax, and classification layers, extract deep features, which are then combined using CCA fusion. The classification of leukemia subtypes is performed using machine learning classifiers, evaluated using metrics like accuracy, sensitivity, specificity, precision, and F1-score. The Bagging Ensemble classifier outperforms others, highlighting the system’s robustness and potential for clinical application. Despite the promising results, the study acknowledged some limitations and future directions. The method’s performance is dependent on high-quality, well-annotated datasets, which may limit its applicability in settings with varied data quality. Additionally, there is a potential risk of overfitting, despite the use of data augmentation techniques. Future research aims to extend the current methodology to include larger, more diverse datasets and refine the algorithms to improve robustness and accuracy. The authors propose further integration of this automated system into clinical workflows, aiding in the early and accurate diagnosis of leukemia, which could significantly enhance patient outcomes. Future research directions include differentiating between more subtypes of leukemia and exploring the applicability of the methodology to other hematologic and non-hematologic conditions.

This vision underscores the potential of advanced deep learning techniques to revolutionize medical diagnostics, improving efficiency and accuracy in detecting malignant conditions. ^33^

Shafique and Tehsin (2018) proposed a robust and automated detection method for acute lymphoblastic leukemia (ALL) and its subtypes (L1, L2, and L3) using transfer learning with a pretrained deep convolutional neural network (DCNN) AlexNet. The ALL-IDB database, which contains images from both healthy patients and leukemia patients, was used for this research. The dataset was split into training and evaluation sets, with data augmentation applied to prevent overfitting. The authors utilized the AlexNet architecture, which includes five convolutional layers followed by three fully connected layers. The last layers were replaced with new ones to classify the input images into four classes: L1, L2, L3, and Normal. Preprocessing steps included image normalization, conversion into binary images, and noise reduction. The feature extraction was done using the pretrained layers of AlexNet, and classification was achieved using the softmax activation function. The model achieved an accuracy of 99.50% for ALL detection and 96.06% for subtype classification. The researchers highlighted that DCNNs are powerful enough to detect and classify leukemia without the need for manual image segmentation. The study also suggests further improvements by integrating the approach into a fully automated system and expanding the dataset for training from scratch to enhance diagnostic accuracy. The methodology involves several key steps. In preprocessing, images are normalized, converted into binary format, and subjected to noise reduction. Unlike traditional methods, no manual segmentation is needed due to the strength of the DCNN layers. Feature extraction is performed using the pretrained AlexNet layers, and the classifier is a modified AlexNet architecture with new fully connected layers for classification. The network includes five convolutional layers, three max-pooling layers, and three newly added fully connected layers for classification. The algorithm characteristics include ReLU activation, softmax for classification, and data augmentation techniques like image rotation and mirroring to enhance the model’s robustness. The study achieved notable performance metrics with an accuracy of 99.50% for ALL detection and 96.06% for subtype classification. Sensitivity was 100% for ALL detection and 96.74% for subtype classification, while specificity was 98.11% for ALL detection and 99.03% for subtype classification. These results underscore the high accuracy and effectiveness of using transfer learning with DCNNs for leukemia detection. Despite the promising results, the study acknowledges some limitations and future directions. The limited dataset could affect the robustness of the training, and further research is needed to integrate the system into a fully automated diagnostic tool. Future work should focus on training deep learning models from scratch with larger datasets to improve accuracy and reliability. The authors also propose integrating their approach into a fully automated system to assist pathologists and oncologists in the early and accurate diagnosis of leukemia, ultimately enhancing patient outcomes through timely treatment interventions. ^34^

Chiaretti et al. (2014) outlined a comprehensive approach to diagnosing and subclassifying Acute Lymphoblastic Leukemia (ALL) using bone marrow morphology, multi-channel flow cytometry (MFC), and genetic/cytogenetic analysis, following the WHO classification of lymphoid neoplasms. Their work provides a detailed overview of current standards and methodologies. The approach integrates cell morphology, immunophenotyping, and genetic/cytogenetic studies as outlined in the 2008 WHO classification. Morphological assessment is the initial diagnostic step, distinguishing ALL from AML based on cell characteristics in bone marrow and peripheral blood. Immunophenotyping with multi-channel flow cytometry (MFC) is the gold standard for identifying cell lineage and defining subsets, utilizing specific markers for B- and T-lineage ALL. Cytogenetic and genetic analyses, including karyotyping, fluorescence in situ hybridization (FISH), array-CGH, and next-generation sequencing (NGS), further refine the diagnosis and provide critical prognostic information.

The article classifies ALL into B-lymphoblastic leukemia/lymphoma not otherwise specified, B-lymphoblastic leukemia/lymphoma with recurrent cytogenetic alterations, and T-lymphoblastic leukemia/lymphoma. Each subtype is characterized by specific immunophenotypic markers and genetic aberrations. For instance, B-lineage ALL markers include CD19, CD20, CD22, CD24, and CD79a, while T-lineage ALL markers include CD1a, CD2, CD3, CD4, CD5, CD7, and CD8. The review highlights the prognostic significance of various genetic lesions, such as IKZF1 deletions in BCR-ABL+ ALL and CRLF2 rearrangements in B-ALL, which are associated with poor outcomes. The authors advocate for prospective clinical trials to ensure accurate diagnosis and effective therapy, emphasizing that early diagnostic work should be performed by experienced personnel to optimize treatment outcomes. ^33^ Despite its strengths, the study acknowledges certain limitations and future directions. The method heavily relies on high-quality samples and advanced diagnostic facilities, which may not be accessible everywhere. Additionally, diagnostic accuracy can vary across different laboratories. To address these issues, the authors stress the importance of conducting early and accurate diagnostic work-ups by experienced personnel. They recommend integrating advanced genetic and immunophenotyping techniques into clinical practice to optimize treatment strategies. Future research aims to identify novel subgroups with prognostic significance and refine diagnostic algorithms to enhance patient outcomes. Prospective clinical trials are also suggested to ensure diagnostic accuracy and therapeutic efficacy, ultimately improving the comprehensive understanding and treatment of ALL. ^35^

Minal D. Joshi, Atul H. Karode, and S.R. Suralkar (2013) developed an automatic method for detecting acute leukemia in blood microscopic images using image processing techniques and a k-nearest neighbor (kNN) classifier. The methodology involves preprocessing, segmentation, feature extraction, and classification to differentiate between normal and leukemic cells. In the preprocessing step, RGB images are converted to grayscale, contrast is enhanced using histogram equalization, and linear contrast stretching is applied to adjust image intensity levels. The segmentation process employs Otsu’s thresholding method to convert grayscale images to binary images, followed by morphological operations to remove small pixel groups and noise. Feature extraction focuses on calculating three morphological features of lymphocyte cells: area, perimeter, and circularity. These features are then used by the kNN classifier to classify the cells as either normal or blast (leukemic). The proposed system was evaluated on 108 images from the ALL-IDB public dataset, achieving an overall accuracy of 93%. The methodology includes several key steps. Preprocessing involves converting RGB images to grayscale, applying histogram equalization for contrast enhancement, and using linear contrast stretching for image intensity adjustment. Noise reduction is achieved through area and closing operations. Segmentation is done using Otsu’s thresholding to convert grayscale images to binary images, followed by morphological operations to remove small pixel groups and noise, and connect neighboring pixels to form objects. Feature extraction calculates the area by counting the total number of non-zero pixels within the nucleus region, measures the perimeter by calculating the distance between successive boundary pixels of the nucleus, and calculates circularity. The kNN classifier then uses these features to classify lymphocyte cells as either normal or blast (leukemic). The performance metrics showed an overall accuracy of 93%, with high precision in classifying blast cells, high recall in identifying leukemic cells, and high specificity in distinguishing between normal and blast cells. Despite the promising results, the study identified some limitations and future directions. The system’s performance depends on high-quality, well-annotated datasets, and it may be sensitive to variations in image quality and staining. Future research aims to optimize image processing techniques to improve robustness and accuracy, integrate more advanced machine learning classifiers, and explore stain-independent segmentation methods to enhance reliability. Expanding the dataset and refining the algorithms to improve the classification of various leukemia subtypes are also recommended. Integrating this automated system into clinical workflows could significantly aid in early and accurate leukemia diagnosis, thereby improving patient outcomes. ^36^

Tusneem A. Elhassan et al. (2023) propose a novel approach for classifying atypical white blood cells (WBCs) in Acute Myeloid Leukemia (AML) using a hybrid model that integrates a geometric transformation (GT) with a deep convolutional autoencoder (DCAE) and a convolutional neural network (CNN). The methodology involves preprocessing, augmentation, feature extraction, and classification to differentiate between typical and atypical WBCs, followed by further subclassification of atypical WBCs into eight distinct categories. The dataset used includes 18,365 single-cell images from AML patients and non-malignant controls, categorized into 15 different types of WBCs.

The preprocessing steps include random rotation, vertical and horizontal flipping, and augmentation using the GT-DCAE model. Feature extraction is performed using the DCAE, which compresses the input data into a low-dimensional latent representation, followed by a CNN for additional feature extraction. The classification involves a two-stage process: the first stage differentiates between typical and atypical WBCs, and the second stage classifies atypical WBCs into subtypes. The proposed model achieved an average accuracy of 97%, a sensitivity of 97%, and a precision of 98%, with an AUC of 99.7%, demonstrating exceptional discriminating abilities. The methodology includes several key steps. Preprocessing involves augmentation techniques like random rotation and flipping, with the GT-DCAE model generating synthetic images. Segmentation is carried out using the CMYK-Moment Localization-Feature Fusion Extraction framework. For feature extraction, the DCAE model employs an encoder network with three convolutional layers, a latent vector space, and a decoder network. Classification is conducted in two stages using CNNs with ReLU activation, L2 regularization, and the SoftMax function.

Performance metrics highlight the model’s effectiveness with an overall accuracy of 97%, precision of 98%, sensitivity of 97%, and an AUC of 99.7%. Class-wise performance indicates high precision and sensitivity for various WBC subtypes, such as myeloblasts and monoblasts, although there are areas for improvement in detecting certain subtypes like metamyelocytes and bilobed promyelocytes.

Despite the promising results, the study identifies some limitations and future directions. The model’s performance depends on high-quality, well-annotated datasets, and there is potential for misclassification in the intermediate stages of myelopoiesis. Future research aims to refine the hybrid model by increasing the dataset size and incorporating additional augmentation techniques. The authors also propose integrating this model into clinical workflows to aid in the early and accurate diagnosis of AML, thereby improving patient outcomes through timely treatment interventions. Furthermore, they suggest exploring the application of their model to other hematologic and non-hematologic conditions to expand its utility and effectiveness in medical diagnostics. ^37^

Negm et al. (2017) present an advanced decision support system for acute leukemia classification using digital microscopic images, combining neural networks and decision tree models to achieve high efficiency and accuracy. The neural network model, favored for its sensitivity, comprises multiple convolutional layers, batch normalization, and dropout to prevent overfitting, while the decision tree model, noted for its speed, provides a robust comparison.

The methodology involves several steps. Preprocessing techniques such as histogram equalization and contrast enhancement are employed to improve image quality. Segmentation is performed using Otsu thresholding and fuzzy c-means clustering. Feature extraction involves capturing geometric, textural, and color features, with Principal Component Analysis (PCA) used for feature reduction. Classification is conducted using support vector machines (SVM) and k-nearest neighbors (KNN), achieving high accuracy rates. The performance metrics reveal that the neural network model achieves an accuracy of 99.74%, demonstrating superior performance in terms of sensitivity, specificity, and precision compared to the decision tree model. However, the study highlights the need for larger datasets and suggests future work on noise reduction and fully automated systems to further enhance the model’s robustness and accuracy.

Despite its strengths, the study identifies some limitations. The limited dataset size may affect the generalizability of the results, and noise reduction methods were not fully explored. Future research aims to develop a robust segmentation system independent of stains, expand the dataset to include more types of acute myeloid leukemia cells, and implement noise reduction methods such as median, mean, and Gaussian smoothing. Additionally, the authors propose enhancing deep learning models to learn from scratch with larger datasets, aiming to create practical, everyday diagnostic tools. ^38^

Niranjana Sampathila et al. (2022) present an advanced method for detecting acute lymphoblastic leukemia (ALL) using a customized convolutional neural network (CNN) named ALLNET. The study utilizes the C_NMC_2019 dataset, containing 10,661 images, with 7272 images of blast cells and 3389 images of healthy cells. The methodology involves extensive preprocessing, including color space conversion, histogram equalization, and thresholding to enhance image quality and facilitate segmentation. Data augmentation techniques such as vertical and horizontal flipping, random rotation, brightness adjustments, and Gaussian blur are employed to increase the dataset’s size and robustness.

Feature extraction and classification are performed using the customized ALLNET architecture, which consists of four convolutional layers, four max-pooling layers, and three fully connected layers, with batch normalization and dropout layers to prevent overfitting. Training was conducted on Google Collaboratory using the Nvidia Tesla P-100 GPU, achieving a maximum accuracy of 95.54%, specificity of 95.81%, sensitivity of 95.91%, F1-score of 95.43%, and precision of 96%. This study underscores the importance of using deep learning models in medical diagnostics to enhance the accuracy and efficiency of leukemia detection. The methodology includes several key steps. Preprocessing involves converting images to the HSI color space for better contrast, applying histogram equalization, and using thresholding to convert images to binary form for segmentation. Noise reduction techniques, including Gaussian blur, are also used. Data augmentation further improves robustness with techniques such as vertical and horizontal flipping, random rotation, brightness adjustments, and Gaussian blur. Feature extraction uses the ALLNET architecture, which includes four convolutional layers with ReLU activation, four max-pooling layers to reduce dimensionality, three fully connected layers with batch normalization and dropout to prevent overfitting, and batch normalization to stabilize and accelerate training. The classification process employs the customized CNN (ALLNET), using the Adam optimizer and categorical cross-entropy loss function. Performance metrics highlight the model’s effectiveness with an accuracy of 95.54%, precision of 96%, sensitivity of 95.91%, specificity of 95.81%, and an F1-score of 95.43%. These results demonstrate the high classification accuracy and robustness of the ALLNET model in detecting leukemia. Despite the promising outcomes, the study identifies some limitations and future directions. The model’s performance depends on high-quality, well-annotated datasets, and it requires extensive preprocessing. Future research aims to enhance the ALLNET model by expanding the dataset to include more diverse and noisy images, reducing preprocessing steps, and integrating more advanced neural network architectures like YOLOv4, ResNet, and AlexNet. These improvements aim to develop a robust and reliable diagnostic tool for clinical use, enhancing the accuracy and efficiency of leukemia detection and reducing the time and error associated with manual diagnostics. ^39^

Pałczyński et al. (2021) developed a hybrid artificial intelligence system for classifying Acute Lymphoblastic Leukemia (ALL) using an optimized, IoT-friendly neural network architecture. This system integrates transfer learning with MobileNet v2 as the encoder and machine learning algorithms like XGBoost, Random Forest, and Decision Tree as classifiers. Utilizing the ALL-IDB2 dataset, which consists of segmented images of lymphocyte cells from healthy patients and patients with ALL, the methodology involves preprocessing the images using data augmentation techniques, encoding them with MobileNet v2, and classifying the feature vectors with the machine learning models. The results demonstrate an average accuracy of over 90%, reaching up to 97.4%, showcasing the effectiveness of hybrid AI systems in handling tasks with low computational complexity. The methodology includes several steps, starting with preprocessing, where RGB images are converted to grayscale, contrast is enhanced using adaptive histogram equalization, and background noise is removed using area and closing operations. Data augmentation techniques such as color jitter, Gaussian blur, horizontal flip, vertical flip, and rotation are applied, followed by Z-score normalization. For feature extraction, MobileNet v2 is utilized, optimized for small processing units like mobile CPUs or IoT devices. The extracted features are then fed into classifiers such as XGBoost, Random Forest, and Decision Tree, trained using Adam optimizer with early stopping after 1000 epochs. The system achieved a classification accuracy of 97.4% when using MobileNet v2 with a fully connected layer, and over 90% accuracy for most models. Performance metrics including precision, recall, specificity, and AUC values were high, indicating robust model performance. The study highlights the system’s strengths in achieving high classification accuracy, efficient preprocessing, and effective use of transfer learning to prevent overfitting, making it suitable for low-resource environments like IoT. Despite these strengths, the study acknowledges some limitations and future directions. The system’s performance depends on high-quality, well-annotated datasets, and it may be sensitive to variations in image quality and staining. The Decision Tree algorithm showed lower performance compared to XGBoost and Random Forest. Future research aims to further optimize the hybrid AI system to enhance its robustness and accuracy, extend its application to other hematologic conditions, and refine the machine learning models. The authors suggest integrating this automated system into clinical workflows, particularly in low-resource environments, to aid in early and accurate leukemia diagnosis. By leveraging transfer learning and IoT-friendly architectures, the system aims to enhance the efficiency and reliability of medical diagnostics. ^40^

Alexandra Bodzas et al. propose an automated method for detecting acute lymphoblastic leukemia (ALL) from microscopic blood smear images, leveraging image processing techniques and machine learning models.

The dataset used in their study consists of 18 images of normal blood smears and 13 images from patients diagnosed with ALL, all collected from the University Hospital of Ostrava. Each image has a resolution of 4,080 × 3,072 pixels, capturing diverse regions of the blood smear to ensure comprehensive representation of leukocytes.

The images underwent preprocessing to correct variations in lighting and staining inconsistencies, followed by segmentation through a three-phase filtering technique to separate leukocytes from the rest of the image. Sixteen morphological and statistical features, mimicking the criteria used by hematology experts, were extracted from the segmented cells. These features were then used to train both a Support Vector Machine (SVM) and an Artificial Neural Network (ANN). While the results were promising, with the SVM achieving 96.72% accuracy and the ANN 97.52%, the limited dataset raises concerns about potential overfitting and the generalizability of the models. Additionally, the method is focused exclusively on the detection of ALL, limiting its applicability to other subtypes of leukemia. ^41^

Nimesh Patel and Ashutosh Mishra proposed an automated method for leukemia detection from microscopic images in their 2015 study. Their approach focuses on extracting textural and morphological features from cells using Gray Level Co-occurrence Matrices (GLCM) along with cell shape analysis. These extracted features are classified using a binary Support Vector Machine (SVM) classifier, enabling the differentiation between leukemic and normal cells with a reported accuracy of 88.24%, based on blood smear images from leukemia patients. The dataset used in the study consists of 100 images from leukemia patients and 100 images from healthy individuals. However, several limitations are associated with this method. The relatively small dataset (only 200 images) raises concerns about the model’s ability to generalize its results to more diverse datasets. Additionally, the approach is limited to general leukemia detection, without addressing specific subtypes such as Acute Lymphoblastic Leukemia (ALL) or Acute Myeloid Leukemia (AML). Another challenge lies in the reliance on GLCM-based feature extraction, which depends on accurate cell segmentation. Achieving precise segmentation can be difficult with variable-quality images, increasing the risk of classification errors. ^42^

Following the discussion of the various studies, below is Table 2, a summarized comparative chart on these studies alongside their methods.

**Table 2.** Comparative overview of automated Leukemia detection techniques and models.

Author, Year	Work	Methods (algorithms used)	Dataset used	Accuracy evaluation	Ref
Warnat-Herresthal et al. (2020)	Scalable Prediction of AML Using Machine Learning	L1-regularized logistic regression (lasso), k-nearest neighbors, linear SVM, linear discriminant analysis, random forests, deep neural networks (DNN)	12029 samples from 105 studies (HG-U133A microarray, HG-U133 2.0 microarray, RNA sequencing)	High accuracy, sensitivity, and specificity; up to 100% in some scenarios	^31^
Loey et al. (2020)	Deep Transfer Learning for Leukemia Diagnosis	Transfer learning with AlexNet, various classifiers (SVM, linear discriminants, decision trees, k-nearest neighbors), fine-tuned AlexNet	Dataset of 2820 images	First model: 99.79% accuracy; Second model: 100% accuracy	^32^
Baig et al. (2022)	Deep Learning Approach to Detect Malignant Leukemia Cells	CNN (two architectures: 19 layers and 15 layers), feature fusion using Canonical Correlation Analysis (CCA), Bagging Ensemble classifier	Dataset of 4150 images	97.04% accuracy (Bagging Ensemble)	^33^
Shafique and Tehsin (2018)	ALL Detection and Classification Using AlexNet	Transfer learning with AlexNet (five convolutional layers, three fully connected layers), data augmentation	ALL-IDB database	99.50% accuracy (ALL detection), 96.06% accuracy (subtype classification)	^34^
Chiaretti et al. (2014)	Diagnosis and Subclassification of Acute Lymphoblastic Leukemia	Morphological assessment, immunophenotyping (multi-channel flow cytometry), genetic/cytogenetic analysis (karyotyping, FISH, array-CGH, NGS)	Not specified	High accuracy in differentiating ALL subtypes using immunophenotyping and genetic analyses	^35^
Joshi et al. (2013)	WBC Segmentation and Classification for Acute Leukemia Detection	k-nearest neighbor (kNN) classifier, Otsu's thresholding, morphological operations	ALL-IDB public dataset (108 images)	93% accuracy	^36^
Elhassan et al. (2023)	Hybrid Model for AML Classification	Two-stage hybrid model (GT-DCAE, CNN), augmentation (random rotation, vertical and horizontal flipping), deep convolutional autoencoder	Dataset of 18365 single-cell images from AML patients and non-malignant controls	97% accuracy, 98% precision, 97% sensitivity, 99.7% AUC	^37^
Negm et al. (2017)	Decision Support System for Acute Leukemia Classification	Neural networks, decision tree, histogram equalization, Otsu thresholding, fuzzy c-means clustering, PCA	Not specified	99.74% accuracy (neural network model)	^38^
Sampathila et al. (2022)	Customized Deep Learning Classifier for ALL Detection	Customized CNN (ALLNET), data augmentation (vertical and horizontal flipping, random rotation, brightness adjustments, Gaussian blur)	C_NMC_2019 dataset (10661 images)	95.54% accuracy, 95.81% specificity, 95.91% sensitivity, 95.43% F1-score, 96% precision	^39^
Pałczyński et al. (2021)	IoT Application of Transfer Learning for ALL Classification	Transfer learning with MobileNet v2, XGBoost, Random Forest, Decision Tree	ALL-IDB2 dataset	97.4% accuracy	^40^
Bodzas et al.	Automated Detection of ALL Using ANN and SVM	ANN, SVM, image preprocessing and segmentation	31 images (18 normal, 13 ALL cases) from the University Hospital of Ostrava	96.72% accuracy (SVM), 97.52% accuracy (ANN)	^41^
Patel and Mishra (2015)	General Leukemia Detection Using Image Processing	Gray Level Co-occurrence Matrix (GLCM) for feature extraction, binary SVM classifier	200 images (100 healthy, 100 leukemia)	88.24% accuracy	^42^

## 7. Discussion

Our article highlights how advances in artificial intelligence (AI) and image processing are transforming the landscape of leukemia diagnostics. Although many studies present highly technical models with excellent performance metrics, their clinical value becomes clearer when examined against the framework of modern classification systems and professional guidelines. The WHO 5th Edition (2022) and the International Consensus Classification (ICC, 2022) have shifted leukemia taxonomy away from morphology alone toward a genetics- and immunophenotype-driven approach, while the NCCN guidelines provide actionable recommendations for risk stratification and therapy selection. AI methods that begin with preprocessing and segmentation, followed by feature extraction and classification, must therefore be evaluated not only on accuracy but also on their ability to generate outputs consistent with these frameworks. For example, models that distinguish myeloblasts from lymphoblasts or recognize hallmark cytogenetic lesions can directly support WHO-5/ICC diagnostic categories. Similarly, systems that flag Philadelphia chromosome–positive ALL or acute promyelocytic leukemia mirror the NCCN pathway where such recognition immediately alters treatment decisions through the initiation of tyrosine kinase inhibitors or ATRA-based therapy.

A coherent synthesis of the literature suggests that the future of AI in leukemia lies not in producing isolated classification accuracies but in providing clinically relevant decision support. Automated tools that triage suspicious smears could accelerate confirmatory testing, while lineage-aware algorithms aligned with WHO-5/ICC enable precise subtype identification. When coupled with NCCN-oriented prompts such as recommending TP53 or IGHV testing in CLL, or molecular milestone tracking in CML these systems can bridge the gap between computational detection and therapeutic action. Nevertheless, challenges remain: most reported models are trained on small, highly curated datasets, are prone to overfitting, and lack robust external validation across centers, staining methods, and demographic groups. Integrating federated learning, stain normalization, and rigorous prospective trials will be essential to ensure reproducibility and real-world reliability.

In conclusion, AI has clear potential to complement existing diagnostic workflows by embedding itself within the logic of WHO-5/ICC classification and NCCN treatment algorithms. Rather than functioning as stand-alone image classifiers, the most impactful systems will be those that deliver explainable, guideline-aligned outputs, support early and accurate detection, and facilitate personalized therapy. By moving from technical performance alone to clinically grounded decision support, AI can play a central role in reducing diagnostic delays, refining prognostic assessment, and ultimately improving outcomes for patients with ALL, AML, CLL, and CML.

## 8. Limitations

AI-powered models have advanced leukemia detection and classification significantly, yet several limitations must be addressed for reliable performance in clinical settings. A key factor influencing model robustness and accuracy is data quality and diversity. Many studies emphasize that high-quality, diverse datasets are essential to counteract variability across different clinical environments. When models are trained on homogeneous, limited datasets, they struggle to generalize well, especially across institutions that use different imaging equipment or patient demographics. This dependency on well-annotated data and the variability of samples across platforms introduce performance inconsistencies, underscoring the need for extensive and varied datasets to reduce overfitting and improve robustness.

Overfitting risks are particularly prominent in models that rely on deep learning, such as convolutional neural networks (CNNs). Despite the use of techniques like dropout layers and normalization, CNN models can tend to memorize training data rather than identify generalizable patterns. This issue is exacerbated when models are trained on data with limited diversity, impacting their effectiveness on new, unseen data. Addressing overfitting requires a combination of data augmentation, transfer learning, and regularization techniques to enhance model adaptability.

Another critical challenge is dataset size constraints. Limited datasets restrict a model’s ability to capture the full range of leukemia variations, impacting classification accuracy. Smaller datasets are also more prone to overfitting, as the model may “memorize” specific details of the training data instead of learning generalizable features. Without access to larger datasets, models may deliver inconsistent results, particularly when applied to data from different patient populations or clinical environments.

Sensitivity to image quality and staining variations presents another challenge, as models can be highly sensitive to inconsistencies in sample preparation. Variations in staining techniques or image quality can affect classification accuracy, and models trained on high-quality samples may struggle to perform in less controlled environments. This sensitivity limits the practical application of AI models in resource-limited settings where high-quality imaging and staining facilities may not be available. Furthermore, automation and intermediate stage classification are areas that require improvement for AI models to be seamlessly integrated into clinical workflows. Challenges in achieving fully automated, robust classification are particularly evident when classifying subtle differences in cell morphology. Models that can accurately distinguish intermediate cell stages are vital, especially for personalized diagnostics, where precise classification is critical for treatment planning. Thus, achieving automation that can handle these complexities is essential for clinical applicability.

Noise reduction and segmentation sensitivity are also important for consistent model performance. Effective noise reduction is essential, as noisy or low-quality images can hinder model accuracy. Segmentation-based approaches, which involve isolating cell features, are especially sensitive to variations in image quality, making them prone to error if the images do not meet certain standards. Optimization of noise reduction methods could therefore improve model reliability.

Finally, broad applicability and real-world implementation remain significant challenges. Models that perform well in research environments may underperform in real-world clinical applications due to differences in clinical workflows, patient demographics, and medical equipment. This dependency on high-quality, well-annotated data makes it difficult for models to adapt to broader clinical settings without compromising accuracy. To make AI models truly viable for clinical use, they must be tested and validated extensively across multiple institutions. Federated learning, which allows models to be trained on data from various sources while preserving privacy, offers a promising approach to enhancing scalability and adaptability.

Overall, addressing these limitations is essential to ensure that AI-driven leukemia diagnostics are reliable, scalable, and adaptable for real-world clinical environments. With improvements in dataset quality and diversity, robust techniques to mitigate overfitting, and optimized workflows for automation, these tools could substantially enhance leukemia diagnostics and patient care.

Table 3 summarizes the main limitations identified in leukemia-related studies.

**Table 3.** Limitations in AI-Based Leukemia Classification and Detection.

Author, Year	Limitations
Warnat-Herresthal et al. (2020)	Performance variability due to differences across studies and platforms; high dependence on data quality and diversity for generalizability.
Loey et al. (2020)	Potential overfitting despite dropout and normalization; high dependence on well-annotated datasets, limiting broad applicability.
Baig et al. (2022)	Dependence on high-quality, well-annotated data; risk of overfitting due to limited dataset variety; limited applicability in real-world settings.
Shafique and Tehsin (2018)	Limited dataset, affecting the model’s robustness; further improvements needed for full automation; dependency on high-quality images.
Chiaretti et al. (2014)	Requires high-quality samples and advanced diagnostic facilities; may not be feasible in resource-limited settings; variability across labs.
Joshi et al. (2013)	Sensitivity to image quality and staining variations; limited dataset size limits generalization potential.
Elhassan et al. (2023)	Dataset quality-dependent; challenges in intermediate stage classification; further refinement needed for broader clinical use.
Negm et al. (2017)	Limited dataset size affects generalizability; noise reduction methods could be further optimized for better performance.
Sampathila et al. (2022)	High reliance on preprocessing and well-annotated datasets; limited generalizability due to constrained dataset variety.
Pałczyński et al. (2021)	Dependent on high-quality data; sensitive to variations in image quality and staining, limiting robustness.
Bodzas et al.	Limited by small dataset size, increasing overfitting risk; model applicability restricted to detecting ALL only.
Patel and Mishra (2015)	Small dataset size limiting generalizability; segmentation-based approach sensitive to variable image quality.

## 9. Conclusion

The integration of artificial intelligence (AI) and advanced image processing techniques has revolutionized the field of leukemia diagnostics, enabling more accurate, rapid, and comprehensive detection of different leukemia subtypes. By automating key processes such as feature extraction, segmentation, and classification, AI-driven models address the inherent challenges of traditional diagnostic methods, improving both efficiency and diagnostic precision. Furthermore, understanding the global epidemiology and associated risk factors of leukemia is vital for the development of targeted screening programs and tailored treatment strategies. Future research should focus on addressing current limitations, such as ensuring high-quality datasets, mitigating overfitting risks, and enhancing the generalizability of AI models. Emphasis should also be placed on international collaboration, ensuring equitable access to advanced diagnostic technologies across regions to bridge healthcare disparities. Ongoing innovation in AI-based diagnostics will play a pivotal role in reducing the global burden of leukemia, fostering early detection, personalized treatment, and ultimately improving patient outcomes. Through focused efforts and interdisciplinary cooperation, the promise of AI in hematologic diagnostics can be fully realized, paving the way for enhanced healthcare delivery and better quality of life for patients worldwide.

### 9.1 Future directions

Future research must focus on overcoming the limitations currently faced by AI-based leukemia diagnostics to develop more reliable, scalable, and generalizable systems. A key priority is the creation of diverse, multi-institutional datasets to address variability in image quality, staining techniques, and patient demographics, ensuring that AI models perform consistently across different clinical environments. Implementing strategies such as data augmentation, transfer learning, and regularization techniques will be essential to mitigate the risk of overfitting and enhance model generalization. Additionally, the integration of multi-modal data combining genetic, clinical, and imaging information can improve diagnostic accuracy and enable personalized treatment strategies. Advancing federated learning frameworks will allow AI models to be trained across multiple institutions while maintaining patient privacy and data security.

Future systems should also emphasize model transparency and interpretability, ensuring that predictions are understandable and actionable by healthcare professionals to build trust and facilitate clinical adoption. Addressing the challenges of scalability is essential, particularly for deploying AI solutions in resource-limited settings, where computational efficiency must be balanced with diagnostic accuracy. Real-world testing and extensive clinical trials will be necessary to validate these models, refining them to accommodate variations in equipment, clinical protocols, and patient populations. International collaboration, supported by equitable access to advanced diagnostic technologies, will play a pivotal role in bridging healthcare disparities and ensuring that the benefits of AI-driven leukemia diagnostics are accessible globally. Through continuous innovation, interdisciplinary cooperation, and targeted efforts, future AI systems can revolutionize leukemia detection and treatment, ultimately improving patient outcomes and reducing the global burden of this hematologic malignancy.

#### Ethics and consent

Ethics and consent were not required.

## Data Availability

Zenodo: Metadata extracted from the Scopus database was used to analyze the countries, and keywords significantly contributing to leukemia research. The focus was on the integration of artificial intelligence in diagnostics, emphasizing global trends, risk factors, and classification strategies for improving detection and treatment: https://doi.org/10.5281/zenodo.14328973. ^43^ The project contains the following underlying data:
-
**AI in Leukemia Research.csv**: Contains metadata extracted from the Scopus database, focusing on the integration of artificial intelligence in leukemia diagnostics, including global trends and classification strategies. -
**AI in Leukemia Research.txt:** A textual description of the metadata and analysis approach for AI applications in leukemia research. -
**Leukemia Types.csv:** Dataset describing various leukemia types with associated risk factors and global research statistics. -
**Risk Factors Keys.txt:** A key file describing the variables and attributes in the “Risk Factors of Leukemia.csv” dataset. -
**Risk Factors of Leukemia.csv:** Comprehensive dataset analyzing risk factors associated with leukemia. Data are available under the terms of the Creative Commons Attribution 4.0 International license (CC-BY 4.0). This study follows the PRISMA 2020 guidelines for systematic reviews to ensure clarity and reproducibility. A detailed PRISMA checklist outlining the methodology, including search strategies, inclusion criteria, and data synthesis, has been uploaded to Zenodo and is accessible at https://doi.org/10.5281/zenodo.14328973. ^43^

## References

[1] Cline MJ : The Molecular Basis of Leukemia. *N. Engl. J. Med.* févr. 1994;330(5):328–336. 10.1056/NEJM199402033300507 8277954

[2] Chow S Buckstein R Spaner DE : A link between hypercholesterolemia and chronic lymphocytic leukemia. *Leuk. Lymphoma.* avr. 2016;57(4):797–802. 10.3109/10428194.2015.1088651 26325342

[3] Xiao W Nardi V Stein E : A practical approach on the classifications of myeloid neoplasms and acute leukemia: WHO and ICC. *J. Hematol. Oncol.* 2024;17(1):56. 10.1186/s13045-024-01571-4 39075565 PMC11287910

[4] Litjens G : A survey on deep learning in medical image analysis. *Med. Image Anal.* déc. 2017;42:60–88. 10.1016/j.media.2017.07.005 28778026

[5] LeCun Y Bengio Y Hinton G : Deep learning. *Nature.* mai 2015;521(7553):436–444. 10.1038/nature14539 26017442

[6] Bouchbika Z : Cancer incidence in Morocco: report from Casablanca registry 2005-2007. *Pan Afr. Med. J.* sept. 2013;16(31):31. 10.11604/pamj.2013.16.31.2791 24570792 PMC3932129

[7] Miranda-Filho A Piñeros M Ferlay J : Epidemiological patterns of leukaemia in 184 countries: a population-based study. *Lancet Haematol.* janv. 2018;5(1):e14–e24. 10.1016/S2352-3026(17)30232-6 29304322

[8] Al-Muftah M Al-Ejeh F : Cancer Incidence and Mortality Estimates in Arab Countries in 2018: A GLOBOCAN Data Analysis. *Cancer Epidemiol. Biomarkers Prev.* déc. 2023;32(12):1738–1746. 10.1158/1055-9965.EPI-23-0520 37733340 PMC10690144

[9] Elbakali R Abdellatif M Smiri Y : EPIDEMIOLOGICAL STUDY OF SOLID AND HEMATOLOGICAL CANCERS AND THEIR RISK FACTORS IN A POPULATION OF 8851 CASES IN MOROCCO. *Int. Med. J. 1994.* déc. 2021;28:6305–6326.

[10] Bispo JAB Pinheiro PS Kobetz EK : Epidemiology and Etiology of Leukemia and Lymphoma. *Cold Spring Harb. Perspect. Med.* janv. 2020;10(6):a034819. 10.1101/cshperspect.a034819 31727680 PMC7263093

[11] Torres-Roman JS : Leukemia mortality in children from Latin America: trends and predictions to 2030. *BMC Pediatr.* nov. 2020;20(1):511. 10.1186/s12887-020-02408-y 33160309 PMC7648388

[12] Huang J : Disease Burden, Risk Factors, and Trends of Leukaemia: A Global Analysis. *Front. Oncol.* juill. 2022;12. 10.3389/fonc.2022.904292 35936709 PMC9355717

[13] Zhang N Wu J Wang Q : Global burden of hematologic malignancies and evolution patterns over the past 30 years. *Blood Cancer J.* 2023;13:82. 10.1038/s41408-023-00853-3 Reference Source 37193689 PMC10188596

[14] Khoury JD : The 5th edition of the World Health Organization Classification of Haematolymphoid Tumours: Myeloid and Histiocytic/Dendritic Neoplasms. *Leukemia.* 2022;36(7):1703–1719. 10.1038/s41375-022-01613-1 35732831 PMC9252913

[15] Sawyers CL : Chronic Myeloid Leukemia. *N. Engl. J. Med.* avr. 1999;340(17):1330–1340. 10.1056/NEJM199904293401706 10219069

[16] Alaggio R : The 5th edition of the World Health Organization Classification of Haematolymphoid Tumours: Lymphoid Neoplasms. *Leukemia.* 2022;36(7):1720–1748. 10.1038/s41375-022-01620-2 35732829 PMC9214472

[17] Burkitt Lymphoma and Burkitt-like Lymphoma : Background, Etiology and Pathophysiology, Epidemiology. *Consulté le.* sept. 2025, 10 septembre 2025.[En ligne]. https://emedicine.medscape.com/article/1447602-overview?form=fpf

[18] Falini B Martino G Lazzi S : A comparison of the International Consensus and 5th World Health Organization classifications of mature B-cell lymphomas. *Leukemia.* 2023;37(1):18–34. 10.1038/s41375-022-01764-1 36460764 PMC9883170

[19] Ghane N Vard A Talebi A : Classification of chronic myeloid leukemia cell subtypes based on microscopic image analysis. *EXCLI J.* 2019;18:382–404. 10.17179/excli2019-1292 31338009 PMC6635720

[20] Shen J : Artificial Intelligence Versus Clinicians in Disease Diagnosis: Systematic Review. *JMIR. Med. Inf.* août 2019;7(3):e10010. 10.2196/10010 31420959 PMC6716335

[21] Litjens G : A survey on deep learning in medical image analysis. *Med. Image Anal.* déc. 2017;42:60–88. 10.1016/j.media.2017.07.005 28778026

[22] Kermany DS : Identifying Medical Diagnoses and Treatable Diseases by Image-Based Deep Learning. *Cell.* févr. 2018;172(5):1122–1131.e9. 10.1016/j.cell.2018.02.010 29474911

[23] Gulshan V : Development and Validation of a Deep Learning Algorithm for Detection of Diabetic Retinopathy in Retinal Fundus Photographs. *JAMA.* déc. 2016;316(22):2402–2410. 10.1001/jama.2016.17216 27898976

[24] Strickland SA : Genotypic and Clinical Heterogeneity Within NCCN Favorable-risk Acute Myeloid Leukemia. *Leuk. Res.* 2018;65:67–73, févr. 10.1016/j.leukres.2017.12.012 29310020 PMC5856485

[25] Shah B : Acute Lymphoblastic Leukemia, Version 2.2024, NCCN Clinical Practice Guidelines in Oncology. *J. Natl. Compr. Canc. Netw.* 2024;22(8):563–576. 10.6004/jnccn.2024.0051 39413812

[26] NCCN Guidelines for Patients: Chronic Lymphocytic Leukemia. *Chronic Lymphocytic Leukemia.* 2025.

[27] CLL: Personalizing 1L Tx|CCO. Consulté le : 16 septembre2025. [En ligne]. https://clinicaloptions.com/activities/oncology/cll:-personalizing-1l-tx/1000010359/content

[28] Chronic Myelogenous Leukemia (CML): Practice Essentials, Background, Pathophysiology. Consulté le : 16 septembre2025. [En ligne]. https://emedicine.medscape.com/article/199425-overview?form=fpf

[29] Habchi Y Bouddou R Aimer AF : Image Classification of Leukemia Cancer Using Wavelet Deep Neural Network. *PRZEGLĄD ELEKTROTECHNICZNY.* mars 2024;1:240–245. 10.15199/48.2024.03.42

[30] Raje C Rangole J : Detection of Leukemia in microscopic images using image processing. *2014 International Conference on Communication and Signal Processing.* avr. 2014; pp.255–259. 10.1109/ICCSP.2014.6949840

[31] Warnat-Herresthal S : Scalable Prediction of Acute Myeloid Leukemia Using High-Dimensional Machine Learning and Blood Transcriptomics. *iScience.* janv. 2020;23(1):100780. 10.1016/j.isci.2019.100780 31918046 PMC6992905

[32] Loey M Naman M Zayed H : Deep Transfer Learning in Diagnosing Leukemia in Blood Cells. *Computers.* juin 2020;9(2):Art. no 2. 10.3390/computers9020029

[33] Baig R Rehman A Almuhaimeed A : Detecting Malignant Leukemia Cells Using Microscopic Blood Smear Images: A Deep Learning Approach. *Appl. Sci.* janv. 2022;12(13):Art. no 13. 10.3390/app12136317

[34] Shafique S Tehsin S : Acute Lymphoblastic Leukemia Detection and Classification of Its Subtypes Using Pretrained Deep Convolutional Neural Networks. *Technol. Cancer Res. Treat.* janv. 2018;17:1533033818802789. 10.1177/1533033818802789 30261827 PMC6161200

[35] Chiaretti S Zini G Bassan R : DIAGNOSIS AND SUBCLASSIFICATION OF ACUTE LYMPHOBLASTIC LEUKEMIA. *Mediterr. J. Hematol. Infect. Dis.* 2014;6(1):e2014073. 10.4084/mjhid.2014.073 25408859 PMC4235437

[36] Joshi MD Karode AH Suralkar PSR : White Blood Cells Segmentation and Classification to Detect Acute Leukemia Ms. 2013. Consulté le: 18 octobre 2024. [En ligne]. Reference Source

[37] Elhassan TA : Classification of Atypical White Blood Cells in Acute Myeloid Leukemia Using a Two-Stage Hybrid Model Based on Deep Convolutional Autoencoder and Deep Convolutional Neural Network. *Diagnostics.* janv. 2023;13(2):Art. no 2. 10.3390/diagnostics13020196 36673006 PMC9858290

[38] Negm A Hassan O Kandil A : A decision support system for Acute Leukaemia classification based on digital microscopic images. *Alex. Eng. J.* oct. 2017;57:2319–2332. 10.1016/j.aej.2017.08.025

[39] Sampathila N : Customized Deep Learning Classifier for Detection of Acute Lymphoblastic Leukemia Using Blood Smear Images. *Healthcare.* oct. 2022;10(10):Art. no 10. 10.3390/healthcare10101812 36292259 PMC9601337

[40] Pałczyński K Śmigiel S Gackowska M : IoT Application of Transfer Learning in Hybrid Artificial Intelligence Systems for Acute Lymphoblastic Leukemia Classification. *Sensors.* déc. 2021;21(23):8025. 10.3390/s21238025 34884029 PMC8659925

[41] Bodzas A Kodytek P Zidek J : Automated Detection of Acute Lymphoblastic Leukemia From Microscopic Images Based on Human Visual Perception. *Front. Bioeng. Biotechnol.* août 2020;8. 10.3389/fbioe.2020.01005 32984283 PMC7484487

[42] Patel N Mishra A : Automated Leukaemia Detection Using Microscopic Images. *Procedia Comput. Sci.* déc. 2015;58:635–642. 10.1016/j.procs.2015.08.082

[43] Achir A : Advances in Leukemia Detection and Classification: A Systematic Review of AI and Image Processing Techniques. *Zenodo.* 27 novembre 2024. 10.5281/zenodo.14328973 PMC12586985 41200664

