# Supplementary material for: Advances in Leukemia detection and classification: A Systematic review of AI and image processing techniques
Source: F1000Res. 2025 Oct 2;13:1536. Originally published 2024 Dec 19. [Version 2] doi: 10.12688/f1000research.159318.2 (PMC12586985; doi:10.12688/f1000research.159318.2)
Supplement: Peer review history

# Reviewer report: Reviewer response for version 2

**DOI:** 10.5256/f1000research.184061.r427792

**Published:** 2025-12-26

**Authors:** Monica Dugaesescu

**Affiliations:** 1 “Carol Davila” University of Medicine and Pharmacy, Bucharest, Romania

I had the pleasure of reviewing the manuscript entitled *Advances in Leukemia detection and classification: A Systematic review of AI and image processing techniques.*

 In the beginning, the manuscript presents thoroughly high-quality statistics of global epidemiology of leukemia and clarifies the leukemia subtypes and their diagnosis for the readers. The results provide a comprehensive overview of the advances in AI-assisted leukemia diagnosis, covering a significant amount of scientific literature published in this field.

 In my opinion, weaknesses of the paper include the fact that it is not clear from the content and the structure of the manuscript the purpose of performing, at the same time, a review on risk factors, leukemia classification, and AI role in image processing for leukemia detection. It is mainly presented as a review of AI applications, though the methods sections and its subsections introduce the research on risk factors and leukemia classification as well, its role in the broader context of the paper being rather unclear.

 The paper does not address any of the limitations of this systematic review. There might be an issue with the first line of section *4. Overview of AI in medical diagnostics*, a part of the phrase or paragraph might be missing.

Is the work clearly and accurately presented and does it cite the current literature?

Yes

If applicable, is the statistical analysis and its interpretation appropriate?

Not applicable

Are all the source data underlying the results available to ensure full reproducibility?

Yes

Is the study design appropriate and is the work technically sound?

Yes

Are the conclusions drawn adequately supported by the results?

Yes

Are sufficient details of methods and analysis provided to allow replication by others?

Yes

Reviewer Expertise:

Laboratory medicine (hematology, biochemistry, immunology, microbiology, molecular biology and genetics), medical bioinformatics

I confirm that I have read this submission and believe that I have an appropriate level of expertise to confirm that it is of an acceptable scientific standard.

# Reviewer report: Reviewer response for version 2

**DOI:** 10.5256/f1000research.184061.r427791

**Published:** 2025-11-22

**Authors:** Vincent Alcazer

**Affiliations:** 1 Hospices Civils de Lyon, Lyon, France

This manuscript presents a broad, PRISMA-guided systematic review and bibliometric analysis of artificial intelligence (AI) applications in leukemia, with a particular focus on image-processing and convolutional neural network (CNN)–based approaches for diagnostic support. The authors also summarise global epidemiology and risk factors, and devote a substantial section to updated classification frameworks (WHO-HAEM5 and ICC 2022) for the main leukemia entities (ALL, AML, CLL, CML).

 The manuscript is timely and broadly informative, particularly in how it links AI applications to up-to-date WHO and ICC 2022 classification schemes. It has clear educational value for hematology clinicians who are not AI specialists.

 There are methodological and scope-related limitations that, in my view, justify additional revision

 - The “systematic review” component relies on a single database (Scopus) without formal risk-of-bias assessment or protocol registration. For a systematic review, this is a significant limitation, particularly in a field where many key AI and hematology papers are indexed in MEDLINE/PubMed and Embase but not always in Scopus. I recommend that the authors explicitly acknowledge the single-database constraint as a methodological limitation in the Methods and Limitations sections. If feasible, extending the search to at least MEDLINE/PubMed would strengthen the systematic nature of the work. Also, please clarify whether any language restrictions were applied.

 - The manuscript does not report a formal risk-of-bias / quality assessment of AI diagnostic studies (e.g. PROBAST, TRIPOD-AI frameworks). Given the high risk of overfitting and spectrum bias in small, single-centre AI studies, this is a crucial missing element. At minimum, the authors should explicitly state that no formal risk-of-bias tool was applied and add a paragraph discussing how this may affect the reliability of reported performance metrics (particularly studies reporting >95–99% accuracy on small datasets).

 - AI coverage is strongly image-centric, with minimal discussion of other clinically mature machine-learning approaches such as models based solely on routine complete blood count (CBC) and laboratory parameters, including large, externally validated tools such as AI-APL (PMID 38670741). Additional presentatin of these approach should be proposed.

 - The current AI section presents an extensive catalogue of individual studies and reported accuracies (often >95–99%), but the synthesis remains largely descriptive. I would encourage the authors to Structure the synthesis by modality and task (e.g. blood smear / image, complete blood count, genomic ... and diagnosis vs outcome), 

 provide a higher-level summary table to precises Typical dataset sizes (n, centres, public vs single-institution), whether external validation was performed and key performance metrics and their clinical relevance (e.g. sensitivity for APL vs non-APL). and integrate Integrate a brief methodological critique. This would move the review from a “who did what” catalogue to a true critical synthesis, more in line with the article’s ambitions. Besides, reporting accuracies of more than 99% in biology should always be criticized. 

 - Other recent reviews in the field might be cited: the recent blood review for example (AI in Hematology, 2025)

Is the work clearly and accurately presented and does it cite the current literature?

Yes

If applicable, is the statistical analysis and its interpretation appropriate?

Not applicable

Are all the source data underlying the results available to ensure full reproducibility?

No source data required

Is the study design appropriate and is the work technically sound?

Yes

Are the conclusions drawn adequately supported by the results?

Yes

Are sufficient details of methods and analysis provided to allow replication by others?

Yes

Reviewer Expertise:

AI, leukemia, myeloproliferative neoplasms, transcriptomic, bioinformatics

I confirm that I have read this submission and believe that I have an appropriate level of expertise to confirm that it is of an acceptable scientific standard, however I have significant reservations, as outlined above.

# Reviewer report: Reviewer response for version 2

**DOI:** 10.5256/f1000research.184061.r423541

**Published:** 2025-11-04

**Authors:** Hamza SEKKAT

**Affiliations:** 1 Hassan First University, Settat, Morocco

The manuscript titled *“Advances in Leukemia detection and classification: A Systematic review of AI and image processing techniques”* is well written, timely and relevant to the current trends in medical AI. It provides a clear and comprehensive synthesis of the literature on artificial intelligence applications for leukemia diagnosis, integrating epidemiological data, classification systems and machine learning approaches.

**Strengths**

- The paper is methodologically solid and follows the PRISMA framework correctly.
- The revision to include the WHO-HAEM5 (2022) and ICC (2022) classification updates adds strong clinical value and makes the review up-to-date.
- The discussion section successfully links AI advances to modern hematopathological classifications and clinical practice guidelines (NCCN).
- The figures and tables are informative and enhance readability.
- The language is clear and accessible for both clinicians and data scientists.

**Minor Points for Improvement**

1. **Clarity and conciseness**: Some paragraphs (especially in the AI section) could be shortened or simplified to improve flow.
2. **Discussion**: Add a short paragraph summarizing key clinical implications and practical recommendations for integrating AI tools in real hospital workflows.
3. **Formatting**: Double-check spacing, alignment and numbering consistency (particularly in multi-column tables).

**Recommendation**

 This is a valuable, comprehensive, and well-organized review that provides important insight into the evolving role of AI in leukemia diagnostics. I recommend **acceptance after minor revision** to improve clarity.

Is the work clearly and accurately presented and does it cite the current literature?

Yes

If applicable, is the statistical analysis and its interpretation appropriate?

Not applicable

Are all the source data underlying the results available to ensure full reproducibility?

Yes

Is the study design appropriate and is the work technically sound?

Yes

Are the conclusions drawn adequately supported by the results?

Yes

Are sufficient details of methods and analysis provided to allow replication by others?

Yes

Reviewer Expertise:

Medical Physics; Artifical Intelligence in Medical Imaging

I confirm that I have read this submission and believe that I have an appropriate level of expertise to confirm that it is of an acceptable scientific standard.

# Reviewer report: Reviewer response for version 2

**DOI:** 10.5256/f1000research.184061.r420820

**Published:** 2025-11-03

**Authors:** Mohammad Al-Agil

**Affiliations:** 1 King's College Hospital NHS Foundation Trust, London, England, UK

This thorough systematic review examines advances in AI and image processing techniques for leukaemia detection and classification. The authors analysed over 25k scientific articles from Scopus using PRISMA, focusing on ALL, AML, CLL, and CML. The review covers global epidemiological trends, risk factors, classification methods, and a comprehensive comparison of AI applications, particularly CNNs, for leukaemia diagnostics. The manuscript provides a detailed bibliometric analysis and evaluates various machine learning and deep learning approaches, discussing their performance metrics, limitations, and clinical applications.

 With regards to the discussion, I believe that it would benefit from an additional brief section to explore how AI tools could be integrate into existing clinical workflows (i.e. at what point would said AI tools be integrated in a hypothetical EPR infrastructure, even as a thought experiment) and the barriers to implementation, ideally citing a real-world example.

 Otherwise, I believe that this manuscript should be indexed.

Is the work clearly and accurately presented and does it cite the current literature?

Yes

If applicable, is the statistical analysis and its interpretation appropriate?

Not applicable

Are all the source data underlying the results available to ensure full reproducibility?

Yes

Is the study design appropriate and is the work technically sound?

Yes

Are the conclusions drawn adequately supported by the results?

Yes

Are sufficient details of methods and analysis provided to allow replication by others?

Yes

Reviewer Expertise:

Natural language processing, predictive ML models, infection prediction, CLL

I confirm that I have read this submission and believe that I have an appropriate level of expertise to confirm that it is of an acceptable scientific standard, however I have significant reservations, as outlined above.

# Reviewer report: Reviewer response for version 2

**DOI:** 10.5256/f1000research.184061.r419584

**Published:** 2025-10-30

**Authors:** Huan Mo

**Affiliations:** 1 National Institutes of Health, Bethesda, Maryland, USA

The current revision has adequately addressed my concerns in the hematological and oncological aspects.

Is the work clearly and accurately presented and does it cite the current literature?

No

If applicable, is the statistical analysis and its interpretation appropriate?

Not applicable

Are all the source data underlying the results available to ensure full reproducibility?

No source data required

Is the study design appropriate and is the work technically sound?

Yes

Are the conclusions drawn adequately supported by the results?

Partly

Are sufficient details of methods and analysis provided to allow replication by others?

Yes

Reviewer Expertise:

NA

I confirm that I have read this submission and believe that I have an appropriate level of expertise to confirm that it is of an acceptable scientific standard.

# Reviewer report: Reviewer response for version 1

**DOI:** 10.5256/f1000research.175028.r354217

**Published:** 2025-01-14

**Authors:** Huan Mo

**Affiliations:** 1 National Institutes of Health, Bethesda, Maryland, USA

As a hematopathologist, this review will be focusing on the the Section 3 (Classification of Leukemia). 

 The FAB morphological classifications used in this manuscript are very dated with limited therapeutic or prognostic values in the current standard of care. Please use the current WHO HAEM5 or ICC classification: 

 WHO Myeloid: [Ref 1]

 WHO Lymphoid (For CLL and ALL): [Ref 2]

 ICC Myeloid and acute leukemia (including AML, CML, and ALL): [Ref 3]

 ICC Lymphoma (for CLL): [Ref 4]

 The four disease groups selected for this manuscript were not a representative or related combination, although they were mostly-known diseases that bear the name "leukemia" (peripheral blood with or without marrow involvement), particularly for the two diseases with the "chronic" title.

 CLL (or CLL/SLL) is a mature B cell lymphoma commonly with leukemic states. Other mature B- and T-cell neoplasms commonly presenting with leukemic state also include mantle cell lymphoma, hairy cell leukemia, splenic marginal zone lymphomas, T-PLL, T- or NK-LGLL, and many other disease entities. 

 CML is a myeloproliferative neoplasm (MPN). Other MPN (such as CNL), MPN/MDS (such as CMML and atypical CML), and myeloid/lymphoid neoplasms with eosinophilia and tyrosine kinase gene rearrangements can also present with leukemic state (or cytosis). 

 For ALL, the L3 now is mostly Burkitt lymphoma (a mature B cell lymphoma), which can present as circulating abnormal "blast"-like cells. 

 For sections 5.2 and 5.3, as well as Figure 7 and 8, it would be helpful if the authors can compare your model with the those in the professional guidelines, such as the NCCN guidelines (nccn.org, free registration needed to download). 

 Although I am not an AI expert, the section 6 appears to be a laundry list of summaries of literature, without necessary integration. Instead, the authors should propose a list of items and use the literature to substantiate.

Is the work clearly and accurately presented and does it cite the current literature?

No

If applicable, is the statistical analysis and its interpretation appropriate?

Not applicable

Are all the source data underlying the results available to ensure full reproducibility?

No source data required

Is the study design appropriate and is the work technically sound?

Yes

Are the conclusions drawn adequately supported by the results?

Partly

Are sufficient details of methods and analysis provided to allow replication by others?

Yes

Reviewer Expertise:

Hematology, medical informatics.

I confirm that I have read this submission and believe that I have an appropriate level of expertise to confirm that it is of an acceptable scientific standard, however I have significant reservations, as outlined above.

## References

[1] : The 5th edition of the World Health Organization Classification of Haematolymphoid Tumours: Myeloid and Histiocytic/Dendritic Neoplasms. *Leukemia* .2022;36(7) : 10.1038/s41375-022-01613-1 1703-1719 10.1038/s41375-022-01613-1 35732831 PMC9252913

[2] : The 5th edition of the World Health Organization Classification of Haematolymphoid Tumours: Lymphoid Neoplasms. *Leukemia* .2022;36(7) : 10.1038/s41375-022-01620-2 1720-1748 10.1038/s41375-022-01620-2 35732829 PMC9214472

[3] : International Consensus Classification of Myeloid Neoplasms and Acute Leukemias: integrating morphologic, clinical, and genomic data. *Blood* .2022;140(11) : 10.1182/blood.2022015850 1200-1228 10.1182/blood.2022015850 35767897 PMC9479031

[4] : The International Consensus Classification of Mature Lymphoid Neoplasms: a report from the Clinical Advisory Committee. *Blood* .2022;140(11) : 10.1182/blood.2022015851 1229-1253 10.1182/blood.2022015851 35653592 PMC9479027
